# Dominant myosin storage myopathy mutations disrupt striated muscles in *Drosophila* and the myosin tail–tail interactome of human cardiac thick filaments

**DOI:** 10.1093/genetics/iyae174

**Published:** 2024-11-01

**Authors:** Meera C Viswanathan, Debabrata Dutta, William A Kronert, Kripa Chitre, Raúl Padrón, Roger Craig, Sanford I Bernstein, Anthony Cammarato

**Affiliations:** Division of Cardiology, Department of Medicine, Johns Hopkins University School of Medicine, 720 Rutland Avenue, Baltimore, MD 21205, USA; Department of Biology, Molecular Biology Institute and Heart Institute San Diego State University, 5500 Campanile Drive, San Diego, CA 92182, USA; Division of Cell Biology and Imaging, Department of Radiology, University of Massachusetts Chan Medical School, 55 Lake Avenue North, Worcester, MA 01655, USA; Department of Biology, Molecular Biology Institute and Heart Institute San Diego State University, 5500 Campanile Drive, San Diego, CA 92182, USA; Division of Cardiology, Department of Medicine, Johns Hopkins University School of Medicine, 720 Rutland Avenue, Baltimore, MD 21205, USA; Division of Cell Biology and Imaging, Department of Radiology, University of Massachusetts Chan Medical School, 55 Lake Avenue North, Worcester, MA 01655, USA; Division of Cell Biology and Imaging, Department of Radiology, University of Massachusetts Chan Medical School, 55 Lake Avenue North, Worcester, MA 01655, USA; Department of Biology, Molecular Biology Institute and Heart Institute San Diego State University, 5500 Campanile Drive, San Diego, CA 92182, USA; Division of Cardiology, Department of Medicine, Johns Hopkins University School of Medicine, 720 Rutland Avenue, Baltimore, MD 21205, USA

**Keywords:** MHC, *MYH7*, assembly competence domain, hyaline inclusions, cardiomyopathy, myosin rod, Genetic Models of Rare Diseases

## Abstract

Myosin storage myopathy (MSM) is a rare skeletal muscle disorder caused by mutations in the slow muscle/β-cardiac myosin heavy chain (MHC) gene. MSM missense mutations frequently disrupt the tail's stabilizing heptad repeat motif. Disease hallmarks include subsarcolemmal hyaline-like β-MHC aggregates, muscle weakness, and, occasionally, cardiomyopathy. We generated transgenic, heterozygous *Drosophila* to examine the dominant physiological and structural effects of the L1793P, R1845W, and E1883K MHC MSM mutations on diverse muscles. The MHC variants reduced lifespan and flight and jump abilities. Moreover, confocal and electron microscopy revealed that they provoked indirect flight muscle breaks and myofibrillar disarray/degeneration with filamentous inclusions. Incorporation of GFP-myosin enabled in situ determination of thick filament lengths, which were significantly reduced in all mutants. Semiautomated heartbeat analysis uncovered aberrant cardiac function, which worsened with age. Thus, our fly models phenocopied traits observed among MSM patients. We additionally mapped the mutations onto a recently determined, 6 Å resolution, cryo-EM structure of the human cardiac thick filament. The R1845W mutation replaces a basic arginine with a polar-neutral, bulkier tryptophan, while E1883K reverses charge at critical filament loci. Both would be expected to disrupt the core and the outer shell of the backbone structure. Replacing L1793 with a proline, a potent breaker of α-helices, could disturb the coiled-coil of the myosin rod and alter the tail–tail interactome. Hence, all mutations likely destabilize and weaken the filament backbone. This may trigger disease in humans, while potentially analogous perturbations are likely to yield the observed thick filament and muscle disruption in our fly models.

## Teaser

Myosin storage myopathy (MSM) is a rare disease caused by mutations that affect myosin’s tail. MSM is characterized by skeletal muscle weakness and occasionally cardiac disease. The L1793P, R1845W, and E1883K MSM mutations are predicted to disrupt and destabilize the human thick filament backbone. Our Drosophila models, expressing a single L1793P, R1845W, or E1883K Mhc allele, phenocopy human disease hallmarks including skeletal muscle dysfunction and cardiomyopathy. Thus, they may serve as a valuable tool for wide-scale testing of therapeutic MSM modalities, which are currently lacking.

## Introduction

Myosins constitute a large superfamily of motor proteins. They convert chemical energy, through ATP hydrolysis, into mechanical work for diverse cellular movements, including cell migration and muscle contraction. The well-studied, conventional class II myosins are hexameric proteins, consisting of 2 myosin heavy chains (MHCs) and 2 pairs of nonidentical light chains (Sweeney *et al.* 2020). The MHC N-terminal, globular S1 head displays the chemo-mechanical, actin-, and nucleotide-binding properties of the holoenzyme. The C-terminal α-helices of the 2 heavy chains intertwine to form an extended coiled-coil rod that terminates in a small nonhelical tail piece.

**Fig. 1.**
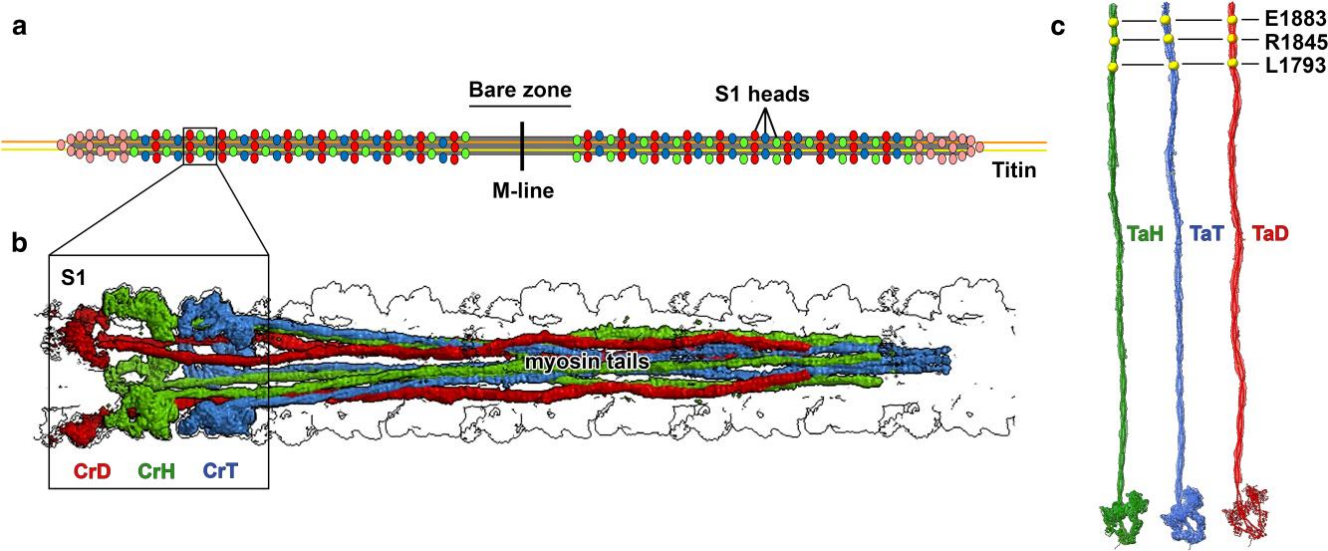
The thick filament and the loci of 3 MSM mutations. a) Illustration of the human cardiac thick filament defining the bare zone, which lacks myosin S1 heads, the central M-line, S1 head pairs (spheres), and titin (yellow, orange). S1 heads are depicted with a quasi-helical arrangement corresponding in color to the “IHMs” that consist of the 2 interacting S1 heads folded back onto the myosin tail, as detailed in Dutta *et al*. (2023). b) Myosin molecules form 3 different types of IHMs that interact with each other, with titin, and with cMyBP-C to dictate filament architecture and function (Dutta *et al.* 2023). Green myosins are displayed forming “crowns” with *Horizontal* IHMs (CrH), blue with *Tilted* IHMs (CrT), and red with *Disordered* IHMs (CrD). c) The MSM mutations L1793P, R1845W, and E1883K (yellow spheres) replace residues within or near the C-terminal ACD of the tail (Sohn *et al.* 1997), which is essential for thick filament formation. The tails corresponding to each IHM crown are colored and labeled accordingly, i.e. green, TaH; blue, TaT; and red, TaD. Figure adapted from Dutta *et al.* (2023).

The coiled-coil rods of sarcomeric myosins self-assemble into bipolar thick filaments in muscle cells, by virtue of a repetitive heptad arrangement of amino acids. The canonical tail sequence consists of 28 recurring residues that establish alternating positively and negatively charged zones. These repeating, clustered zones facilitate electrostatic interactions with residues of staggered neighboring rods and other myosin-binding proteins, resulting in the ordered formation of thick filaments (McLachlan and Karn 1982). In addition, the rod's 29-residue assembly competence domain (ACD) possesses a unique charge “fingerprint” with 4 central negative residues flanked by 2 distinct positive blocks (Sohn *et al.* 1997). The basic residues in the ACD are essential for tail–tail interactions and filament assembly (Ricketson *et al.* 2010). Myosins polymerize into filaments by initially assembling in an antiparallel fashion, followed by the parallel incorporation of molecules on each opposing filament half. Hence, the mature bipolar thick filament is characterized by a headless bare zone located at its center (Fig. 1a; Craig and Woodhead 2006). Recently determined cryo-EM and cryo-ET structures resolved the complex protein–protein interactome of human [Protein Data Bank (PDB): 8G4L] (wwPDB Consortium 2019) and murine cardiac thick filaments, respectively (Dutta *et al.* 2023; Tamborrini *et al.* 2023). The thick filament models define numerous head-to-head and tail-to-tail contacts, as well as cMyBP-C and titin interactions with tails that are vital for the form and regulated function of the biomolecular machine (Dutta *et al.* 2023).

*MYH7* is located on the long arm of chromosome 14 (14q11.2) and encodes beta myosin heavy chain (β-MHC). β-MHC is found primarily in the adult heart and slow (type I) skeletal muscle fibers. The ∼23 kb *MYH7* gene contains 40 exons that direct the synthesis of the 1935 amino acid β-MHC isoform. The gene is important clinically, as it is associated with over 500 mutations that engender diverse diseases (Colegrave and Peckham 2014). These include hypertrophic cardiomyopathy-1 (HCM; OMIM #192600) (Online Mendelian Inheritance in Man 2024), dilated cardiomyopathy-1S (OMIM #613426), left ventricular noncompaction cardiomyopathy (OMIM #604169), Laing distal myopathy (OMIM #160500), autosomal dominant (OMIM #608358) and recessive (OMIM #255160) myosin storage myopathy (MSM), and subgroups of congenital myopathies with characteristic histopathological features such as multiminicores and myofiber type disproportion with small type I fibers (Oldfors 2007).

MSM is a rare protein aggregate myopathy with <90 cases reported to date. It was initially described in 1971 as “familial myopathy with probable lysis of myofibrils of type I fibers” (Cancilla *et al.* 1971), and then called “hyaline body myopathy” in 1994 due to the glassy appearance of the inclusion bodies (Engel 1986). Subsequently, MSM was referred to as “scapuloperoneal or limb-girdle myopathy” based on the muscles involved in the disease (Masuzugawa *et al.* 1997). Tajsharghi *et al.* (2003) introduced the term “MSM,” because of the intense immunoreactivity of the inclusions' contents to slow/β-cardiac MHC antibodies. Genotypic analyses have identified 10 *MYH7* MSM-causing mutations in the C-terminal region of the tail, namely R1845W (Tajsharghi *et al.* 2003), H1901L (Bohlega *et al.* 2004), L1793P (Dye *et al.* 2006), E1883K (Tajsharghi *et al.* 2007), L1779P (Chai *et al.* 2007), X1936W (Ortolano *et al.* 2011), K1784del (Stalpers *et al.* 2011), R1820W (Yuceyar *et al.* 2015), X1936L (Bánfai et al. 2017), and R1712W (Beecroft *et al.* 2019); the I457R mutation (Mamelona *et al.* 2019) occurs in the myosin head. The 3 MSM mutations investigated in this study (L1793P, R1845W, and E1883K) replace highly conserved residues in myosin's distal rod region (Supplementary Fig. 1), within or near the ACD. Their locations along the coiled-coil tails are shown in Fig. 1b and c.

MSM patients exhibit considerable heterogeneity in presentation. First symptoms can manifest as either young-onset (Cancilla *et al.* 1971; Barohn *et al.* 1994; Tajsharghi *et al.* 2003; Bohlega *et al.* 2004; Laing *et al.* 2005) or as adult forms (Barohn *et al.* 1994; Masuzugawa *et al.* 1997; Bohlega *et al.* 2003, 2004; Tajsharghi *et al.* 2003, 2007; Laing *et al.* 2005; Rafay *et al.* 2005; Shingde *et al.* 2006; Pegoraro *et al.* 2007; Uro-Coste *et al.* 2009; Yuceyar *et al.* 2015). The pattern of inheritance can be sporadic (Tajsharghi *et al.* 2003; Stalpers *et al.* 2011) or familial with autosomal dominant (Masuzugawa *et al.* 1997; Bohlega *et al.* 2003, 2004; Tajsharghi *et al.* 2003; Shingde *et al.* 2006; Pegoraro *et al.* 2007; Uro-Coste *et al.* 2009) or recessive-affiliated (Tajsharghi *et al.* 2007; Yuceyar *et al.* 2015; Beecroft *et al.* 2019) phenotypes. The clinical signs commonly include hypotonia with delayed motor milestones, symmetrical proximal muscle weakness in both the upper and lower limbs (Barohn *et al.* 1994; Pegoraro *et al.* 2007) and scapuloperoneal dystrophy with shoulder girdle weakness, referred to as “winging of scapula” with or without pseudohypertrophy of calves and foot drop (Oldfors *et al.* 2005; Shingde *et al.* 2006). Nonprogression or rapid progression with variations in severity, within afflicted families (Bohlega *et al.* 2004; Uro-Coste *et al.* 2009), have also been noted. Cardiac disease in the form of ventricular/septal hypertrophy (Pegoraro *et al.* 2007), HCM (Tajsharghi *et al.* 2007; Uro-Coste *et al.* 2009), neonatal cardiomyopathy (Uro-Coste *et al.* 2009), and cardiac failure (Önengüt *et al.* 2004) has been reported in certain MSM patients. MSM is distinguished by subsarcolemmal hyaline bodies in type I fibers, which is a disease hallmark (Barohn *et al.* 1994; Masuzugawa *et al.* 1997; Bohlega *et al.* 2003; Tajsharghi *et al.* 2003; Laing *et al.* 2005; Rafay *et al.* 2005; Shingde *et al.* 2006; Chai *et al.* 2007; Pegoraro *et al.* 2007; Uro-Coste *et al.* 2009; Stalpers *et al.* 2011), with unaffected type II fibers (Tajsharghi *et al.* 2003). Electron microscopy has additionally shown accumulations of nonmembrane bound granular/filamentous material within the myofibers (Barohn *et al.* 1994; Masuzugawa *et al.* 1997; Bohlega *et al.* 2003; Tajsharghi *et al.* 2003; Laing *et al.* 2005; Shingde *et al.* 2006; Supala-Berger *et al.* 2009; Uro-Coste *et al.* 2009; Stalpers *et al.* 2011).

*Drosophila melanogaster* has benefited research endeavors for over a century and, for the past 3 decades, has been employed as a model organism to study an increasing number of human diseases. *Drosophila* is an invaluable tool to study myosin-based muscle diseases as it possesses a single muscle *Mhc* gene that relies solely on alternative RNA splicing for different isoform expression in distinct muscles (Bernstein *et al.* 1983; George *et al.* 1989). In addition, *Drosophila* thoracic indirect flight muscle (IFM) fibers are exceptionally well-ordered and are comprised of myofibrils analogous to those of humans (Reedy and Beall 1993). These traits, in conjunction with readily available IFM and jump muscle MHC-null (O'Donnell *et al.* 1989; Collier *et al.* 1990) and complete MHC-null (O'Donnell and Bernstein 1988) organisms, make flies an ideal animal model to study various myosin myopathies (Wang *et al.* 2012; Suggs *et al.* 2017; Viswanathan, Tham, *et al.* 2017; Dahl-Halvarsson *et al.* 2018, 2020; Rao *et al.* 2019). We previously established *Drosophila* MSM models that were homozygous for MSM-causing *Mhc* alleles in nonessential IFM and jump muscles (Viswanathan, Tham, *et al.* 2017). The mutants showed skeletal muscle and myofibrillar structural and functional defects with hyaline inclusions that recapitulated human disease. Our studies additionally demonstrated that mutant myosins purified from these flies displayed impaired in vitro filament assembly properties and/or decreased filament stability. Furthermore, another recent report showed a dose-dependent correlation of disease severity in *Drosophila* MSM models (Dahl-Halvarsson *et al.* 2020).

For the present study, we used transgenic MSM *Drosophila* to create animals that were heterozygous for the mutant MHCs (MSM/+), to more faithfully model disease dominance as observed in patients. We found that flies expressing 1 copy of the *L1793P*, *R1845W*, or *E1883K* MSM-causing alleles exhibited significantly reduced lifespans. The mutants displayed flight and jump impairment, associated with disrupted skeletal muscle, myofibrillar, sarcomeric, and thick filament architecture, and an accumulation of extrasarcomeric myosin aggregates. Cardiomyopathy as well as cardiac myofibrillar defects were also observed. Further, MSM mutations mapped on the cryo-EM-derived human cardiac thick filament atomic model revealed likely disruptions in tail–tail interactions in the filament backbone, which would be expected to compromise overall thick filament integrity. Thus, weakened filament stability likely contributes to MSM pathogenesis in humans and muscle dysfunction in our fly models.

## Materials and Methods

### Generation of transgenic *Drosophila*

*L1793P*, *R1845W*, and *E1883K Mhc* mutant *Drosophila* were generated using the *P* element-mediated germline transformation method (Spradling and Rubin 1982) as previously described (Viswanathan, Tham, *et al.* 2017). Transgenic lines were maintained either in the *yw* background, harboring 2 endogenous wildtype (*Mhc*^*+*^) and 2 transgenic (*Mhc*^▾^) copies of *Mhc* (i.e. *Mhc*^*+*^*/Mhc^+^; Mhc*^▾^*/Mhc*^▾^) or in the IFM and jump muscle, MHC-null *Mhc*^*10*^ background (i.e. *Mhc*^*10*^*/Mhc^10^; Mhc*^▾^*/Mhc*^▾^; O'Donnell *et al.* 1989). The latter, therefore, expresses exclusively transgenic *Mhc*^▾^ in IFM and jump muscle, and both *Mhc*^*+*^ and *Mhc*^▾^ in all remaining musculature. To ensure phenotypes were not influenced by differences in myosin dosage, transgenic MHC abundance in IFMs was determined to be comparable to wildtype in all transgenic lines (Viswanathan, Tham, *et al.* 2017). Appropriate alternative splicing of transgenic *Mhc* transcripts was also verified. To mitigate concerns related to the effects of transgene cytolocation on phenotype, multiple transgenic lines per mutant genotype were investigated. Three *L1793P* lines (*L1793P-1*, *L1793P-4*, and *L1793P-6*), 4 *R1845W* lines (*R1845W-3*, *R1845W-6*, *R1845W-2*, and *R1845W-1*), and 4 *E1883K* lines (*E1883K-2*, *E1883K-5*, *E1883K-3*, and *E1883K-4*) were used for further studies (see Table 1 for transgene chromosomal location). The transgenic wildtype *PwMhc2* (Swank *et al.* 2000) line served as a control.

**Table 1.** Transgene chromosomal locations (Viswanathan, Tham, *et al.* 2017) and serial flight indices of the second cohort of heterozygous transgenic lines.

Transgenic line	Transgene cytolocation	Flight index 1d mean ± SEM (*n*)	Flight index 2d mean ± SEM (*n*)	Flight index 7d mean ± SEM (*n*)
*PwMhc2/+*	X	5.17 ± 0.09 (210)	5.04 ± 0.11 (221)	4.75 ± 0.12 (258)
*L1793P-1/+*	4	1.23 ± 0.08 (221)	1.00 ± 0.07 (218)	0.41 ± 0.06 (220)
*L1793P-4/+*	4	1.31 ± 0.08 (213)	0.99 ± 0.07 (219)	0.42 ± 0.06 (214)
*L1793P-6/+*	X	1.26 ± 0.08 (217)	1.01 ± 0.08 (224)	0.35 ± 0.06 (208)
*R1845W-3/+*	3	1.17 ± 0.07 (220)	1.08 ± 0.08 (218)	0.51 ± 0.06 (217)
*R1845W-6/+*	3	1.34 ± 0.13 (218)	1.04 ± 0.08 (213)	0.65 ± 0.07 (214)
*R1845W-2/+*	3	1.16 ± 0.07 (210)	0.96 ± 0.07 (227)	0.54 ± 0.07 (209)
*R1845W-1/+*	3	1.26 ± 0.08 (213)	1.00 ± 0.07 (223)	0.41 ± 0.06 (211)
*E1883K-2/+*	3	1.22 ± 0.07 (215)	1.02 ± 0.08 (215)	0.22 ± 0.04 (223)
*E1883K-3/+*	2	1.19 ± 0.08 (196)	1.08 ± 0.07 (220)	0.40 ± 0.06 (208)
*E1883K-4/+*	2	1.25 ± 0.08 (208)	1.13 ± 0.08 (210)	0.41 ± 0.06 (215)
*E1883K-5/+*	3	1.17 ± 0.08 (207)	0.97 ± 0.08 (223)	0.46 ± 0.06 (210)

To breed true heterozygotes, containing only one endogenous and one transgenic *Mhc* copy in *all* tissues, flies expressing 2 native and 2 transgenic *Mhc* copies (*Mhc*^*+*^*/Mhc^+^; Mhc*^▾^*/Mhc*^▾^) were crossed with complete MHC-null *Mhc*^*1*^ flies carrying a balancer chromosome (O'Donnell and Bernstein 1988). The progeny (*Mhc*^*+*^*/Mhc^1^; Mhc*^▾^) were used for longevity and cardiac analyses. To generate flies heterozygous for the MSM mutations solely in IFMs and jump muscles, virgin transgenic females in the *Mhc*^*10*^ background (*Mhc*^*10*^*/Mhc^10^; Mhc*^▾^*/Mhc*^▾^) were crossed with *yw* males, and female offspring (*Mhc*^*+*^*/Mhc^10^; Mhc*^▾^) were collected for longevity, jump, flight, and thoracic imaging studies. In addition, reciprocal crosses were executed using virgin females that expressed 2 native and 2 transgenic *Mhc* copies (*Mhc*^*+*^*/Mhc^+^; Mhc*^▾^*/Mhc*^▾^) and *Mhc*^*10*^ males to generate a second set of offspring, again heterozygous for the mutant MHC in IFMs and jump muscles (*Mhc*^*+*^*/Mhc^10^; Mhc*^▾^). Progeny bred from this scheme were used to corroborate flight test results obtained from the previous mating scheme. Female flies were selected for all studies, as male transgenic flies with 1 copy of the transgene on the X chromosome might have decreased transgenic MHC due to possible lack of dosage compensation (Muller 1932; Mukherjee and Beermann 1965). Flies heterozygous for GFP-tagged MHC and transgenic MHC in the IFMs (i.e. *Mhc*^*GFP*^*/Mhc^10^; Mhc*^▾^) were created by crossing transgenic virgin females in the *Mhc*^*10*^ background (*Mhc*^*10*^*/Mhc^10^; Mhc*^▾^*/Mhc*^▾^) with homozygous *GFP-MHC* (Bloomington Stock Center #50881) males. All fly stocks were maintained on a standard cornmeal–yeast–sucrose–agar medium at 25°C.

### Lifespan analysis

Female flies (*Mhc*^*+*^*/Mhc^1^; Mhc*^▾^ and *Mhc*^*+*^*/Mhc^10^; Mhc*^▾^) were collected on the day of eclosion and aged in uncrowded vials (25 flies per wide vial) with food, at room temperature. Survivors were counted and flipped onto fresh food vials every 2–3 days, until there were no surviving flies. For each line, 2 or 3 independent cohorts of flies (*n* = 100/cohort) were examined. Survival was analyzed using the 2-tailed log-rank (Mantel–Cox) test.

### Jump and flight tests

Newly eclosed female flies (*Mhc*^*+*^*/Mhc^10^; Mhc*^▾^) with clipped wings were aged 1 day at room temperature and subjected to jump tests. Individual flies were placed atop an inverted vial (95 mm high) centered on a sheet of paper within several 0.5 cm-spaced concentric circles and coaxed to jump with a paintbrush. The 3 farthest jump distances, out of 10 trials per fly, were recorded and averaged for 50 flies per genotype (Swank *et al.* 2002). Measured values were logarithmically transformed to ensure normal distribution of all sample sets. Significance was assessed via 1-way ANOVA with Tukey's multiple comparison tests.

Flight tests were performed on 1-, 2-, and 7-day-old female *Drosophila* as described previously (Drummond *et al.* 1991) at room temperature. Each fly was released into the center of a Plexiglas chamber with a light source positioned at the top, and assigned a score of 6 for upward, 4 for horizontal, 2 for downward, or 0 for no flight (Tohtong *et al.* 1995). The average flight index (FI) from 196 to 258 flies was calculated by dividing the sum of the individual scores by the number of animals tested per cohort. Significance was assessed using 2-way ANOVAs with Bonferroni multiple comparison tests. Large sample populations tested assuaged concerns due to heterogeneity in variance within the sample sets.

### Fluorescence microscopy

Pathohistological characterization of the IFMs, using fluorescence microscopy, was performed on *Mhc*^*+*^*/Mhc^10^; Mhc*^▾^ pupae and 1-day-old adults as described previously (Nongthomba and Ramachandra 1999; Viswanathan *et al.* 2015; Viswanathan, Schmidt, *et al.* 2017). Briefly, pupae and young anesthetized flies with heads and abdomens removed were fixed overnight in 4% formaldehyde in 1× PBST at 4°C and rinsed several times in 1× PBST. Fixed specimens were positioned on a glass slide, snap frozen in liquid nitrogen and immediately bisected along the midsagittal plane using a razor blade. Bisected pupae and hemi-thoraces were stained with Alexa-568 Phalloidin (1:100 in 1× PBST, Invitrogen—listed in reagent table) overnight at 4°C and rinsed with 1× PBS before imaging with an EVOS FL cell imaging system (Life Technologies) at 4× magnification.

### Confocal microscopy

Confocal imaging of IFMs was performed on *Mhc*^*GFP*^*/Mhc^10^; Mhc*^▾^ pupae and 1-day-old adult *Drosophila*. Fixed pupae and adult thoraces were oriented for sagittal sectioning in cryo-embedding medium (Tissue-Tek O.C.T. compound) and frozen overnight. Frozen samples were cut on a cryo-microtome, operating between −15 and −18°C, with a section thickness of ∼20 μm, placed on polarized slides, mounted, and imaged using a Zeiss LSM880-Airyscan super-resolution single-point, laser scanning confocal microscope, at 63× magnification. Thick filament lengths were measured using ImageJ. Significance was assessed via the Kruskal–Wallis 1-way ANOVA with Dunn's post hoc test.

For thick filament signal averaging, confocal micrographs of IFM myofibrils were imported into ImageJ. Individual myofibrils were straightened to fill a rectangular box by drawing a segmented line of width equal to the control myofibril width, and executing the “Straighten” command. Single fluorescent A-bands were cropped and combined into an image stack. Images were aligned in *x*- and *y*-coordinate space using the plugin “Template Matching → Align Slices in Stack” (https://sites.google.com/site/qingzongtseng/template-matching-ij-plugin). A reference image was selected, and all images in the stack were translated to align in approximately the same position as the reference. Following automated image alignment, composite thick filament images were generated by executing the “Z Project” command with “average intensity” projection type. Dimensions were assessed by comparing intensity line plots of the averaged fluorescent thick filament signals.

For confocal imaging of *Drosophila* hearts, 1-week-old *PwMhc2/+*, *L1793P/+*, *R1845W/+*, and *E1883K/+* (*Mhc*^*+*^*/Mhc^1^; Mhc*^▾^) cardiac tubes were prepared as detailed in earlier studies (Alayari *et al.* 2009). Semiintact hearts were stained with rabbit anti-*Drosophila* muscle myosin antibody (1:500, kind gift from Dr. Dan Kiehart, Duke University) overnight at 4°C and Cy5-goat antirabbit secondary antibody. Samples were rinsed in 1× PBS, mounted with Vectashield, and visualized using a Leica TCS SPE RGBV confocal microscope (Leica Microsystems) at 40× magnification.

### Transmission electron microscopy

TEM of IFMs was carried out as previously described (O'Donnell and Bernstein 1988). Late-stage (P12–P13) female pupae (*Mhc*^*+*^*/Mhc^10^; Mhc*^▾^) and thoraces from adult females <12 h old were treated with primary fixative (3% paraformaldehyde and 2% glutaraldehyde in 100 mM sodium phosphate pH 7.2) overnight at 4°C, and then secondary fixative (1% osmium tetroxide in 100 mM sodium phosphate pH 7.2) for 2 h at 4°C. Samples were rinsed with distilled water, dehydrated with an acetone series, oriented, and embedded in Spurr's resin, with increasing concentrations of resin from 75% to 100%, before placing into molds and cured at 60°C overnight. Thin sections were cut using a glass or diamond knife and stained with 2% uranyl acetate followed by lead citrate. Images were acquired on an FEI Tecnai 12 transmission electron microscope using a TVIPS 214 high-resolution camera.

### Cardiac physiological analysis

One-, three-, and five-week-old, female *PwMhc2/+*, *L1793P/+*, *R1845W/+*, and *E1883K/+* (*Mhc*^*+*^*/Mhc^1^; Mhc*^▾^) semiintact *Drosophila* hearts (*n* = 35–46) were prepared under artificial hemolymph as previously described (Vogler and Ocorr 2009). Cardiac performance was assessed as formerly outlined (Cammarato *et al.* 2015) and cardiac output was measured (Blice-Baum et al. 2017). Significant differences between genotype and age, and interaction effects, were determined using 2-way ANOVAs with Bonferroni multiple comparison tests.

### MSM mutation mapping on the human cardiac thick filament structure

The L1793P, R1845W, and E1883K mutations were mapped onto the atomic model of a 430 Å repeat of the cardiac thick filament C-zone, where cMyBP-C binds (PDB 8G4L), and on individual full-length myosin molecules using ChimeraX (Pettersen *et al.* 2021) by selecting the residues 1793, 1845, and 1883 on TaH, TaT, and TaD tails. Inside the structure, neighboring tails at the mutation loci were shown by surface charge representation to visualize the local environment and possible interactions. Full-length myosin molecules are the segmented cryo-EM density map fitted with atomic models as described in Dutta *et al*. (2023).

### Statistical analysis

All statistical analyses were performed using GraphPad Prism 10 software. Data are presented as mean ± SEM. Individual statistical tests performed are detailed in each method subsection above. Significance was assessed at *P* < 0.05.

## Results

### MSM mutations decrease lifespan of transgenic flies

**Fig. 2.**
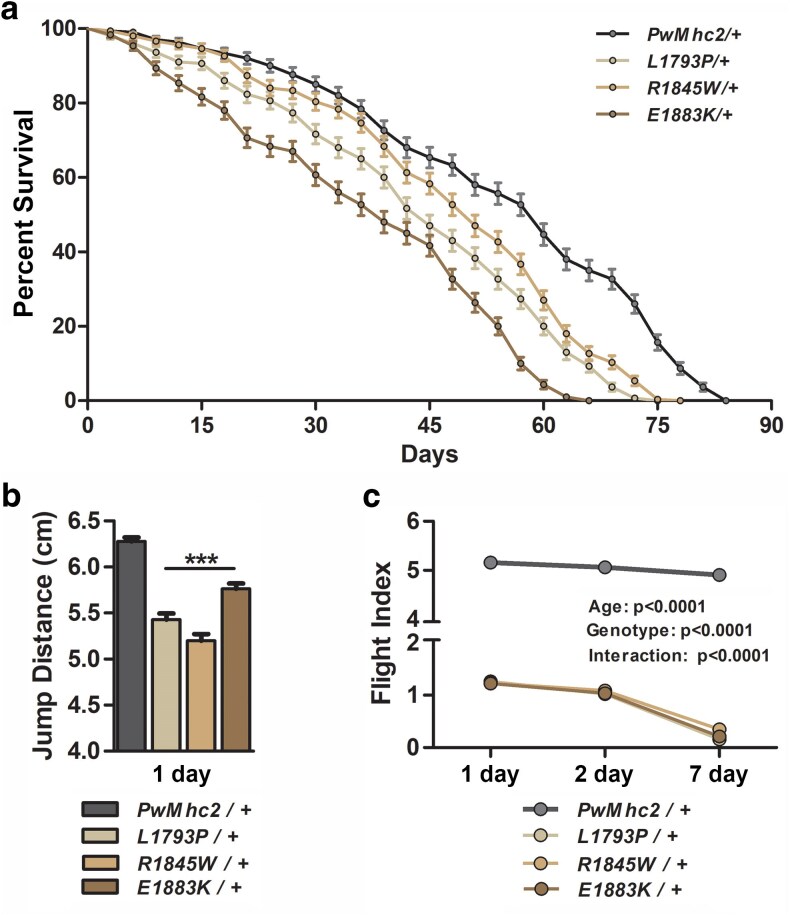
Heterozygous MSM mutations reduce *Drosophila* lifespan and skeletal muscle function. a) A reduction in lifespan in all 3 mutant heterozygotes (*L1793P/+*, *R1845W/+*, and *E1883K/+*) vs *PwMhc2/+* controls was observed (*n* = 300 per genotype). Median and maximum lifespans were both considerably shorter in all mutants (see Results). Comparison of survival curves revealed that the reduction in longevity was significant between the control and each mutant (*P* < 0.0001). b) Jump distances of all heterozygous mutants were significantly reduced relative to the control (***P < 0.0001). This verifies that all 3 mutant alleles act in a dominant negative fashion. c) Flight indices of 1-, 2-, and 7-day-old heterozygous MSM mutants show pronounced reduction relative to age-matched *PwMhc2/+* transgenic controls (genotype, *P* < 0.0001). Moreover, age-related flight deterioration (age, *P* < 0.0001) was markedly more severe in the mutants in accordance with the progressive nature of the disease (interaction, *P* < 0.0001). Results of these locomotion studies verified increasingly impaired mutant skeletal muscle function with age. Data are presented as mean ± SEM.

We previously established that *L1793P*, *R1845W*, and *E1883K Drosophila Mhc* transgenes were homozygous lethal in the *Mhc*^*1*^ null background, i.e. expression of mutant myosin exclusively, throughout *all* musculature, was insufficient for survival (Viswanathan, Tham, *et al.* 2017). To ascertain if heterozygous expression of MSM-disease alleles affected longevity, we analyzed lifespan of transgenic flies in the *Mhc*^*1*^ mutant background, which expressed myosin from a single copy of endogenous wildtype and from a single copy of transgenic *Mhc* (*Mhc*^*+*^*/Mhc^1^; Mhc*^▾^). MSM mutant *Mhc* expression had a significant impact on posteclosion longevity, with a decline in median age from 60 days in the *PwMhc2/+* controls to 39–51 days in the heterozygous mutants (Fig. 2a). Additionally, maximum lifespan of all mutant heterozygotes was significantly reduced relative to *PwMhc2/+* controls (66–78 days vs 84 days; Fig. 2a). Survival of flies in the *Mhc*^*10*^ background, crossed with the *yw* strain (i.e. *Mhc*^*+*^*/Mhc^10^; Mhc*^▾^), with 1 copy of mutant and 2 copies of endogenous wildtype *Mhc* in all muscles *other* than IFM and jump muscles, were also evaluated. The reductions in median survival (48–51 vs 69 days) and maximum lifespan (69–72 vs 87 days) relative to control were remarkably similar to the former cohort, confirming an effect on longevity despite varying mutant to wildtype gene dosage in non-IFM/jump muscles (Supplementary Fig. 2a).

### *L1793P*, *R1845W*, and *E1883K Mhc* mutations act in a dominant negative fashion and depress skeletal muscle function

To determine if our models showed depressed skeletal muscle function, we first carried out jump tests on 1-day-old transgenic heterozygous flies. Virgin females, homozygous for the transgenic wildtype or mutant *Mhc* in the *Mhc*^*10*^ background (*Mhc*^*10*^*/Mhc^10^; Mhc*^▾/^*Mhc*^▾^), were crossed with *yw* males, as above, to generate *Drosophila* expressing an endogenous wildtype and a single *Mhc* transgene (*Mhc*^*+*^*/Mhc^10^; Mhc*^▾^) in their IFM and jump muscles. The average jump distance of 1-day-old *PwMhc2*/+ controls was 6.28 ± 0.04 cm (Fig. 2b). On the contrary, *Drosophila* expressing MSM mutant *Mhc* had significantly diminished jump abilities (5.01–5.83 cm, *P* < 0.0001; Fig. 2b). Multiple transgenic lines of each mutant showed similar reductions in jump ability, thus alleviating concerns due to genetic variability attributable to a different transgene insertion site (Supplementary Fig. 2b).

To evaluate the mutation-induced effects on a second distinct skeletal muscle type, we performed flight tests on 1-, 2-, and 7-day-old female flies produced as described above, for jump analysis. *PwMhc2*/+ transgenic controls displayed normal flight behavior at all ages studied with the majority flying upward (FI of 5.07 ± 0.10, 5.06 ± 0.11, and 4.90 ± 0.11 at 1-, 2-, and 7-days, respectively; Fig. 2c). In contrast, all MSM mutant lines demonstrated a significant reduction in flight ability, with the majority showing downward flight (FI = 1.19–1.28) at 1-day of age, and a near complete lack of flight ability by 7-days of age (FI = 0.44–0.64; Fig. 2c, Supplementary Fig. 2c). In addition, older flies expressing *L1793P*, *R1845W*, or *E1883K Mhc* frequently demonstrated a “wings-up” phenotype that is associated with damaged or “hypercontracted” IFM fibers (Kronert *et al.* 1995; An and Mogami 1996). To confirm that dominant effects were not influenced by genetic background, a second set of heterozygous *Drosophila* (*Mhc*^*+*^*/Mhc^10^; Mhc*^▾^) was examined by crossing virgin females homozygous for transgenic *Mhc* in the *yw* background (*Mhc*^*+*^*/Mhc^+^; Mhc*^▾/^*Mhc*^▾^) with male *Mhc*^*10*^ flies. Flight indices of 1-, 2-, and 7-day-old mutant flies of this cohort showed a strikingly similar reduction in flight ability relative to control (Table 1). The reduced skeletal muscle performance among the heterozygous MSM lines indicates that all 3 mutant *Mhc* alleles act in a dominant negative manner.

### Weakened flight in heterozygous MSM flies is associated with damaged IFM fibers and disrupted myofibrillar structure

**Fig. 3.**
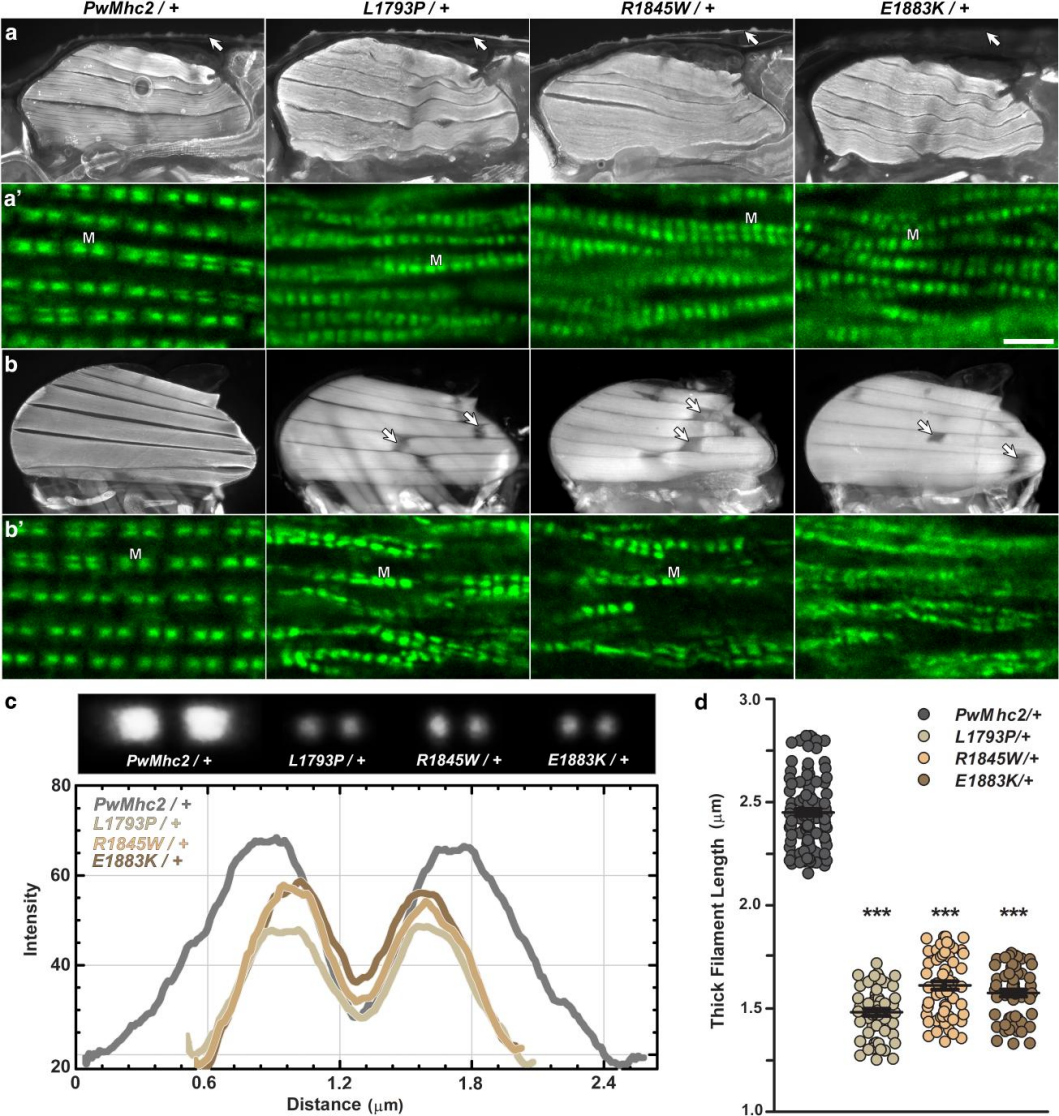
Heterozygous MSM mutations result in torn IFM fibers, diminished myofibrillar and sarcomeric integrity, and shorter thick filaments. a) Fluorescent micrographs of pupal transgenic control (*PwMhc2/+*) and mutant IFMs reveal the presence of 6 DLMs in all genotypes. Arrows delineate the enveloping pupal case. a′) Confocal micrographs of cryo-sectioned *PwMhc2/+* pupae show uniform, parallel IFM myofibrils and discrete sarcomeres with the S1-tagged GFP-MHC forming distinct A-bands with bare zones (M). All 3 mutant heterozygotes had fewer evident myofibrils and thick filaments relative to controls. Furthermore, thick filament lengths appeared smaller among all mutants. Scale bar = 2.5 µm. b) Fluorescent micrographs of 1-day-old adult *PwMhc2/+ Drosophila* show normal IFM morphology, while all 3 heterozygous mutants displayed torn fibers. Arrows point to breaks in the DLMs. b′) Confocal images of cryo-sectioned 1-day-old adult *PwMhc2/+* thoraces reveal ordered myofibrils and thick filaments. However, DLMs from 1-day-old mutants exhibit disarranged myofibrils, with diffuse GFP-labeled MHC localization and fewer distinct sarcomeres and thick filaments. c) Top: Composite images of pupal IFM thick filament GFP signals from (a′). Bottom: line scans of averaged composite GFP signals demonstrate reduced filament lengths in all MSM mutants. d) Pupal thick filament lengths were significantly shorter in all 3 mutant heterozygotes vs *PwMhc2/+* control (***P < 0.0001). Data are presented as scatter plots that display the mean ± SEM for each genotype (*n* = 53–108).

*Drosophila* IFMs are highly sensitive to mutations (Beall and Fyrberg 1991; Bing *et al.* 1998) and are exceptionally well-ordered making them ideally suited for imaging and structural analyses. To investigate if reduced muscle function in the mutants was associated with altered muscle morphology, we imaged IFMs of heterozygous flies (*Mhc*^*+*^*/Mhc^10^; Mhc*^▾^), using fluorescence microscopy. Micrographs of bisected late-stage pupae revealed the presence of intact dorsal-longitudinal IFMs (DLMs) in *PwMhc2/+* control and all 3 MSM mutants, with the 6 fibers spanning the length of the thorax (Fig. 3a). As observed previously in IFM/jump muscle homozygotes (Viswanathan, Tham, *et al.* 2017), heterozygosity did not apparently alter fiber formation. To ascertain if IFM structure worsened posteclosion, we also examined DLM morphology of young adults. One-day-old *PwMhc2/+* flies demonstrated a well-arranged DLM structure (Fig. 3b). All adult MSM heterozygotes, however, exhibited substantial IFM histopathological disruptions relative to late-stage pupae, with torn, hypercontracted DLMs pulling away from break points (Fig. 3b).

Subsequently, we employed confocal microscopy to examine finer IFM thick filament, sarcomeric, and myofibrillar structural details. *Drosophila* pupae, heterozygous for GFP-tagged wildtype *Mhc* and transgenic *Mhc* (*Mhc*^*GFP*^*/Mhc^10^; Mhc*^▾^), were used to visualize thick filament formation and dimensions in situ. Note that the GFP moiety is fused to the N-terminal S1 head and thus likely had little effect on thick filament formation. Confocal micrographs of whole mount pupal IFMs showed conspicuous differences in myofibrillar and in thick and thin filament order between mutant heterozygotes and control (Supplementary Fig. 3). Additionally, super-resolution confocal imaging of pupal thoraces prepared via cryo-sectioning indicated that the mutant sarcomeres appeared less crystalline-like and were characterized by seemingly shorter thick filaments (Fig. 3a′). While micrographs from sectioned adult *PwMhc2/+* DLMs revealed regular sarcomeres and distinct fluorescent A-bands (Fig. 3b′), thoraces of all 3 MSM mutant heterozygotes showed disarranged and fewer myofibrils with abnormal sarcomeres and scarcer A-bands (Fig. 3b′). The myofibrillar and sarcomeric architectures of young mutant adults showed extensive degeneration compared to those of pupae.

### MSM mutations result in shorter thick filaments, in vivo

In vitro assembly assays using purified L1793P, R1845W, and E1883K MHCs from IFM/jump muscle homozygous flies demonstrated a decreased propensity for the mutant myosins to polymerize relative to wildtype MHC, with L1793P filaments exhibiting shorter lengths (Viswanathan, Tham, *et al.* 2017). Altered in vivo thick filament lengths may, therefore, be associated with MSM pathogenesis. GFP-labelled A-bands of randomly selected myofibrils from super-resolution confocal micrographs of *Mhc*^*GFP*^*/Mhc^10^; Mhc*^▾^ pupal muscles were aligned in ImageJ to generate average composite thick filament images (Fig. 3c, top). Intensity line scans of average composite signals of each genotype implied considerably reduced thick filament lengths in all MSM heterozygotes relative to control (Fig. 3c). To quantify the extent of reduction, we measured thick filament lengths directly from micrographs of fluorescing pupal muscles. Average thick filament lengths of all 3 mutants (L1793P: 1.48 ± 0.02 µm; R1845W: 1.61 ± 0.02 µm; E1883K: 1.58 ± 0.02 µm) were significantly shorter vs transgenic controls (*PwMhc2*: 2.45 ± 0.02 µm; *P* < 0.0001; Fig. 3d). Thus, aberrant in vivo thick filament length likely contributes to mutation-mediated pathology.

### Dominant MSM mutations result in myofibrillar ultrastructural defects

**Fig. 4.**
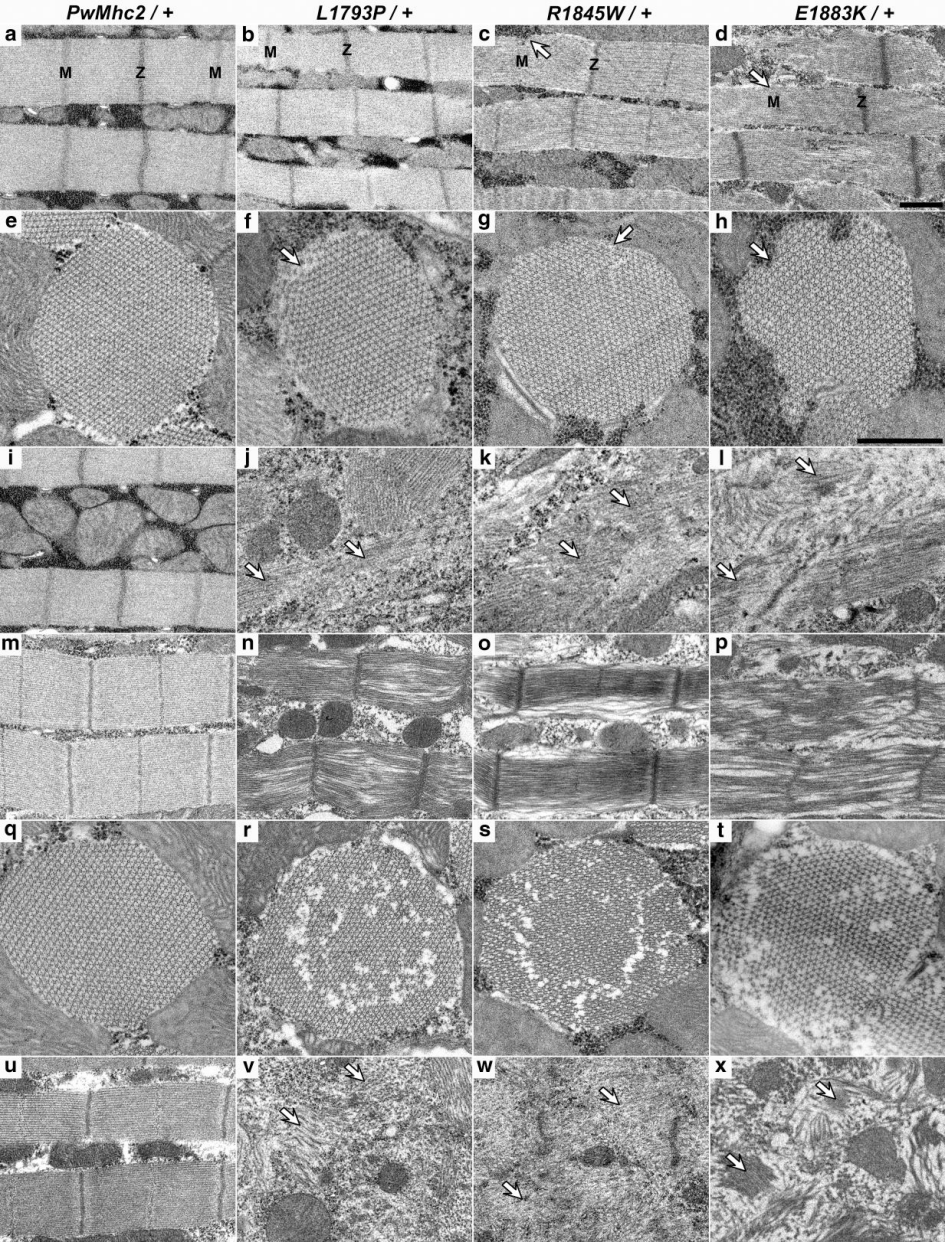
Heterozygous MSM mutations cause IFM myofibrillar ultrastructural defects. TEM micrographs of late-stage pupal (a–l) and young adult (m–x) IFMs. Longitudinal sections of *PwMhc2/+* DLMs showed parallel myofibrils and normal sarcomeric structure with evident Z- and M-lines (a, i, m, u). Transverse sections of *PwMhc2/+* controls displayed crystalline-like double-hexagonal myofilament arrays in pupal (e) and young adult (q) muscles. Longitudinal sections of all 3 mutant pupal DLMs also exhibited predominantly normal myofibrils with a few sarcomeres showing mild myofilament mispacking (b–d, marked with arrows). Cross sections of pupal DLMs of mutant heterozygotes revealed normal myofibrillar core assembly, with some breaks and fissures in the myofilament lattice along the periphery (f–h, marked with arrows). Areas of filamentous inclusions were seen (marked with arrows) within the myofibrils, reminiscent of hyaline bodies in afflicted patients (j–l). Micrographs of DLMs of young adults revealed more apparent defects relative to pupae. Longitudinal sections of all mutants displayed more disordered thick filament arrangements with few identifiable M-lines (n–p). Transverse sections demonstrated several fractures with missing thick filaments resulting in disordered myofilament lattices (r–t). Large areas of vastly disrupted myofibrils and copious granular and filamentous hyaline-like inclusions (marked by arrows) were present (v–x). The ultrastructural defects worsened with age in the mutants. Magnification is the same in all longitudinal sections (a–d, i–l, m–p, u–x). Magnification is the same in all transverse sections (e–h, q–t). Scale bar = 1 µm. Z and M denote Z-lines and M-lines, respectively.

To determine the ultrastructural effects of heterozygous MSM *Mhc* expression on muscle organization and maintenance, we examined the DLMs of late-stage pupae (*Mhc*^*+*^*/Mhc^10^; Mhc*^▾^) and young adults (<12 h old) by transmission electron microscopy. Longitudinal and transverse sections of *PwMhc2/+* controls showed normal assembly and integrity of myofibrils within the IFM. Parallel myofibrils and uniform sarcomeric myofilament arrangement, with easily discernable Z-discs and M-lines, were observed in longitudinal sections, both in the pupal stage and in young adults (Fig. 4a, i, m, and u). Cylindrical myofibrils with a highly ordered, crystalline, double-hexagonal filament lattice (Fig. 4e and q) that is characteristic of *Drosophila* IFMs, were seen in transverse sections. IFMs of transgenic MSM heterozygotes showed minimal ultrastructural defects in the pupae. Longitudinal sections revealed relatively normal sarcomeric structures on average, with a few myofibrils showing small alterations in M-line architecture (Fig. 4b, c, and d—denoted by arrows). Notably, however, some areas of pupal muscle displayed relatively normal sarcomere lengths, as depicted in the images, whereas overall the sarcomeres appeared shorter, consistent with what was observed via confocal microscopy (Fig. 3). Cross sections demonstrated a semblance of normal myofibrillar assembly, with some gaps in the myofilament lattice and occasional fraying along the myofibril periphery (Fig. 4f, g, and h—denoted by arrows). Areas of granular and filamentous inclusions were observed, reminiscent of human hyaline bodies (Fig. 4j, k, and l—denoted by arrows).

Thin longitudinal sections of DLMs in young mutant adults revealed noticeable defects, including disarrayed thick filament arrangement, with loss of identifiable M-lines (Fig. 4n, o, and p). Transverse sections illustrated disruptions of the myofilament lattice. Fissured myofibrils with missing thick filaments were resolved in all 3 mutant heterozygotes (Fig. 4r, s, and t). Numerous areas of highly disrupted myofibrils, composed largely of granular and filamentous hyaline-like inclusions, were also identified (arrows Fig. 4v, w, and x).

### *L1793P*, *R1845W*, and *E1883K* heterozygous MSM mutations perturb cardiac structure and performance

**Fig. 5.**
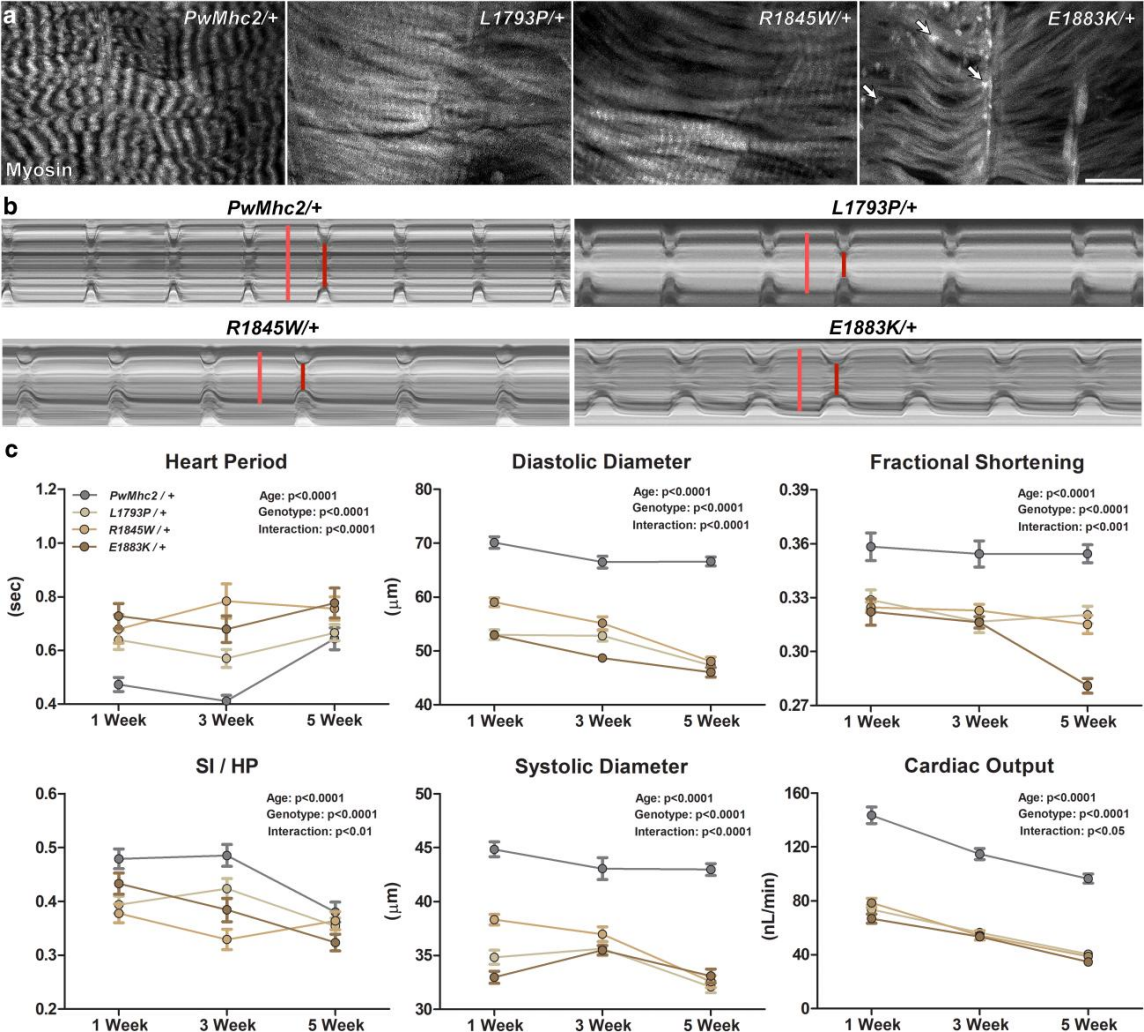
Heterozygous MSM mutations alter cardiac myofibrillar architecture and physiology. a) Myofibrils from the anterior conical chamber of 1-week-old hearts stained with anti-*Drosophila* MHC antibody. *PwMhc2/+* controls displayed organized, spiraling myofibrils and highly structured repetitive striations, indicative of well-ordered sarcomeres. In contrast, hearts of age-matched mutants demonstrated aberrant myofibrils with diffuse cross striations, indicative of compromised myofibrillar maintenance and degeneration. Scale bar = 10 μm. Arrowheads point to extrasarcomeric anti-MHC positive punctae observed in some hearts. b) M-mode kymograms generated from high-speed videos of beating, 1-week-old *PwMhc2/+*, *L1793P-1/+*, *R1845W-6/+*, and *E1883K-2/+* hearts. The traces reveal movements of the heart tube edges in the *y*-axis over a 5 s time period along the *x*-axis. Vertical lines delineate cardiac diameters (light red: diastolic diameter; dark red: systolic diameter). c) *L1793P/+*, *R1845W/+*, and *E1883K/+ Drosophila* exhibited highly significant alterations in several cardiac functional parameters relative to *PwMhc2/+* transgenic control flies. The mutants showed a small yet significant increase in heart period and decrease in SI/HP, indicative of altered cardiac cycle dynamics. Restrictive cardiac physiology, characterized by decreased diastolic and systolic diameters, fractional shortening, and cardiac output, was observed in the mutants at all ages studied. Data are presented as mean ± SEM (*n* = 35–46 for each genotype and age group).

Given the robust expression of *MYH7* in adult human myocardium, MSM mutations occasionally provoke cardiomyopathy in addition to skeletal myopathy (Önengüt et al. 2004; Tajsharghi *et al.* 2007; Uro-Coste *et al.* 2009). Therefore, *Drosophila* hearts were imaged to investigate potentially dominant pathophysiological myocardial effects caused by the MSM *Mhc* alleles. Adult flies possess a 1-mm-long heart tube composed of a single layer of ∼80 cardiomyocytes. Heterozygotes were generated as detailed above by crossing flies with 2 native and 2 transgenic *Mhc* alleles (*Mhc*^*+*^*/Mhc^+^; Mhc*^▾/^*Mhc*^▾^) with complete, *Mhc*^*1*^ MHC-null flies carrying a balancer chromosome (yielding *Mhc*^*+*^*/Mhc^1^; Mhc*^▾^; O'Donnell and Bernstein 1988). Confocal micrographs of opposing cardiomyocytes from the anterior conical chamber of 1-week-old *PwMhc2/+* control hearts showed highly ordered myofibrils with myosin localized into well-organized, discretely striated contractile units (Fig. 5a). On the contrary, *Drosophila* hearts expressing the MSM-disease alleles displayed anomalous cellular and myofibrillar morphology. Specifically, mutant *Mhc* expression resulted in deviant, less discrete, and diffuse localization of myosin within the sarcomeres. Occasionally extrasarcomeric, myosin-immunopositive punctae were identified (arrows, Fig. 5a).

The effects of mutant *Mhc* expression on heterozygote cardiac contractile properties were assessed using high-speed video microscopy and motion analysis software. M-mode kymograms, which illustrate heart wall movement over time, were characterized by mutation-induced reductions in cardiac dimensions and altered contractile dynamics in 1-week-old flies relative to controls (Fig. 5b). To track the progression of these changes over time, we filmed beating cardiac tubes at 1-, 3-, and 5-weeks of age. In accordance with previously published studies (Blice-Baum et al. 2017; Viswanathan, Schmidt, *et al.* 2017), heart period, which is the total duration of a cardiac cycle and is inversely proportional to heart rate, significantly increased with age for all genotypes (Fig. 5c). However, relative to control, all 3 mutants showed slightly prolonged heart periods at all age points studied (Fig. 5c, Supplementary Fig. 4) and, hence, a small, yet significant decrease in the systolic interval/heart period (SI/HP) ratio over time (Fig. 5c). Cardiac physiology of a second cohort of heterozygous flies, derived from different parental lines, showed similarly altered indices (Supplementary Fig. 4). Together, the reduced myogenic heart rate and diminished systolic duration suggest perturbed contractile dynamics in all mutant *Drosophila* hearts. Additionally, cardiac dimensions, fractional shortening, and cardiac output progressively decreased with age (Blice-Baum et al. 2017; Viswanathan, Schmidt, *et al.* 2017) in all lines (Fig. 5c, Supplementary Fig. 4). *Drosophila* expressing mutant *Mhc* demonstrated significantly restricted diastolic and systolic diameters (Fig. 5c, Supplementary Fig. 4) vs control. The effect on diastolic diameters was greater than that on systolic diameters, which resulted in a significant decrease in fractional shortening relative to *PwMhc2/+*. This reduction in cardiac dimensions, in addition to the reduced heart rate, resulted in diminished cardiac output compared to control (Fig. 5c, Supplementary Fig. 4).

### MSM mutations disrupt tail–tail interactions in the human cardiac thick filament

**Fig. 6.**
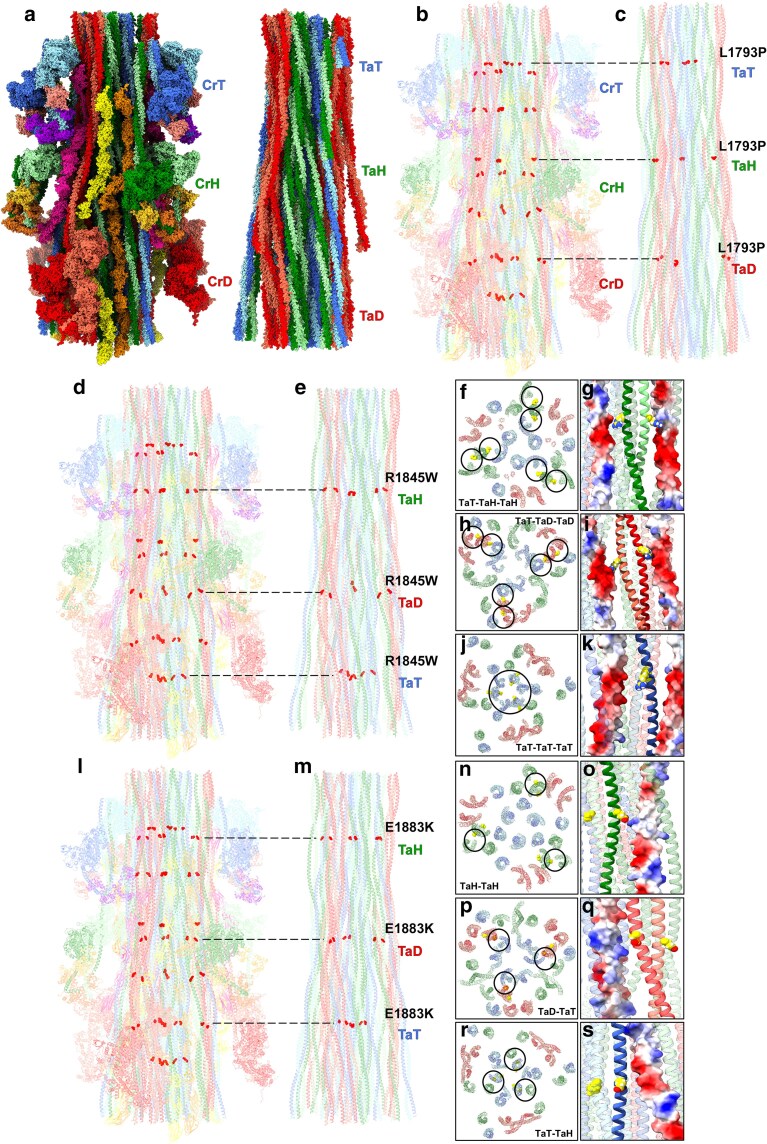
Mapping of the 3 MSM mutations onto the cryo-EM-derived model (PDB 8G4L) of the human thick filament C-zone (Dutta *et al.* 2023). a) Left: Atomic model of a 430 Å repeat of the thick filament C-zone (longitudinal surface view) showing IHMs forming 3 types of crowns (disordered/mobile, CrD, red; tilted, CrT, blue; horizontal, CrH, green) with the M-line oriented at the top. cMyBP-C molecules are displayed in magenta and the 2 titin strands are shown in yellow and orange. Right: filament backbone illustrating longitudinally arranged coiled-coil myosin tails forming an extensive interacting network; S1 heads, titin, and cMyBP-C were removed for clarity. TaD (red), TaH (green), and TaT (blue) denote the myosin tails associated with each type of corresponding crown. b) Translucent atomic model of one 430 Å repeat, showing the 3 crowns of myosin heads, with colors corresponding to (a). Red spheres represent the loci of the L1793P MSM mutation among the 3 tail types. c) Backbone of myosin tails with black dotted lines indicating the position of the L1793P mutation in TaD, TaH, and TaT. d, e) Atomic model as in (b, c) with black dotted lines and red spheres denoting loci of the R1845W mutation among the 3 types of tails. Transverse (f, h, j) and longitudinal (g, i, k) close-up views show the mutation's (yellow) potential effects on different tails due to the disruption of distinct R1845-mediated tail–tail interactions. In (g, i, k), red surface = negative, blue =positive. l, m) Atomic model as in (b, c) with black dotted lines and red spheres specifying the loci of the E1883K mutation along TaD, TaH, and TaT. Transverse (n, p, r) and longitudinal (o, q, s) close-up views show the mutation's (yellow) potential effects on neighboring tails due to the disruption of distinct E1883-mediated tail–tail points of contact. Note: the thick filament atomic model used for mapping is based on a 6 Å-resolution cryo-EM reconstruction. Amino acid side chain locations shown are, therefore, not precise and should be considered as possible locations and interactions as possible interactions.

The 3 MSM mutations were mapped onto the cryo-EM structure of the human cardiac thick filament C-zone (Dutta *et al.* 2023). The cryo-EM map (Fig. 1b) and corresponding atomic model (PDB 8G4L) of the C-zone 430 Å repeat (Fig. 6a) show 3 crowns of myosin interacting-heads motifs (IHMs), each with a different conformation: Horizontal (CrH, green), Tilted (CrT, blue), and Disordered/mobile (CrD, red; Dutta *et al.* 2023). Within the filament backbone, myosin tails originating from these motifs (TaH, TaT, and TaD, respectively; Figs. 1b and 6a) form an extensive interacting network. The L1793P mutation introduces a proline, a known helix breaker, inside the α-helices of the myosin coiled-coil tail (Fig. 6b and c). This may distort the normal paths of the tails, possibly disrupting tail–tail interactions needed to stabilize the native filament. The R1845W mutation replaces a positively charged Arg with a neutral Trp, at critical contact sites of TaH, TaT, and TaD (Fig. 6d–k). This would alter the local surface charge of myosin tails, weakening charge attraction with neighboring tails (Fig. 6g, i, and k). Thus, the stabilizing electrostatic tail–tail interactions (Fig. 6f, h, and j, circles) and overall rigidity of the filament backbone are likely weakened. Moreover, substituting a smaller residue with a bulkier one may introduce local steric hindrance at tail–tail interfaces, which could further weaken the interactome. The E1883K mutation (Fig. 6l, m, n, p, and r) replaces an acidic Glu with a basic Lys, reversing the local surface charge of tails (Fig. 6o, q, and s), and likely weakening electrostatic tail–tail interactions (Fig. 6o and q) and thick filament rigidity. However, the mutation may not directly weaken all TaT-mediated interactions (Fig. 6r and s). Thus, both R1845W and E1883K substitutions alter charges at critical loci and are predicted to disrupt the core and outer shell of the filament backbone.

## Discussion

The United States definition of a rare disease is one that affects fewer than 200,000 individuals or has a prevalence of 1–8 in 10,000 (Aronson 2006). More than 7,000 rare diseases collectively affect over 25 million Americans (Boat and Field 2011). Some of these orphan diseases are extremely uncommon and reported cases are under 100. Such small numbers of affected patients pose a host of challenges that complicate diagnosis and treatment. Developing disease-modifying therapies, that utilize a big-picture perspective while targeting the underlying pathology, remains difficult. Small, inexpensive, genetically pliable, phenotypically accurate model organisms, including *D. melanogaster*, may support this cause.

Recently, for example, 2 studies explored the potential of *Drosophila Abba* (a ubiquitin ligase of the TRIM family) overexpression to modulate muscle dysfunction in fly models of *MYH7-*mediated pathology. Dahl-Halvarsson *et al.* (2018) established a K1729del Laing's distal myopathy fly model, which exhibited dose-dependent disease severity. Muscle-specific overexpression of *Abba* improved muscle function and morphology. In a second study, the authors overexpressed embryonic *Drosophila* MHC (WT or R1845W) to produce MSM-like phenotypes (Dahl-Halvarsson *et al.* 2020). Simultaneous muscle-specific overexpression of *Abba* again tempered muscle pathology, with partial restoration of function. While this latter report corroborates *Abba*'s therapeutic potential, the use of the embryonic MHC isoform to investigate disease in adult flies, introduces confounding effects. Hence, the need for additional, complementary models to study MSM pathogenesis and treatment options persists.

Previously, studies that attempted to resolve the pathomechanisms induced by MSM mutant myosin in situ, in developing cultured human myoblasts, showed certain variants (R1845W and H1901L) resulted in disrupted myofibrillogenesis with myosin aggregates, while others (L1793P) did not (Dahl-Halvarsson *et al.* 2017). *C. elegans* that expressed wildtype and mutant MSM myosin showed compromised motility, likely due to myosin overdose. Nevertheless, the functionality of these transgenic myosins was demonstrated by partial rescue of paralyzed MHC B (*MHC7*) null worms (Dahl-Halvarsson *et al.* 2017). Our previously published study (Viswanathan, Tham, *et al.* 2017) was the first to report, unambiguously, the effects of the L1793P, R1845W, and E1883K *Mhc* mutations, in vivo. We provided evidence of muscle functional defects with myofilamentous inclusions in *Drosophila* muscles expressing exclusively mutant MHC in amounts similar to wildtype strains and transgenic controls (i.e. homozygotes). Our in vitro experiments (Viswanathan, Tham, *et al.* 2017), using purified full-length myosin, also substantiated and supplemented previous studies using MSM LMM fragments to analyze the biochemical features of the disease (Armel and Leinwand 2009).

Here, we analyzed wildtype and MSM mutant flies as true heterozygotes that more closely reflect human genetic composition. While the first reported patient with the E1883K mutation was homozygous, the 2 other affected siblings were not genotyped and, hence, heterozygosity could not be discounted (Tajsharghi *et al.* 2007). This was the first reported mutation with cardiomyopathy in addition to the distinguishing skeletal muscle phenotype that led to the characterization of the disease. The severity of the phenotype and copresentation of cardiac and skeletal myopathy could well be due to the presence of 2 mutant copies of *MYH7.* Considering the high degree of conservation, however, and the indisputable role of electrostatic interactions in maintaining tail–tail contacts in the thick filaments, the severe Glu to Lys charge reversal may likely be the underlying cause of myocardial involvement. Notably, despite being the predominant myosin isoform expressed in the heart, not all MSM-causing *MYH7* mutations engender human cardiomyopathy. In general, *MYH7*-associated disorders are exceptionally heterogenous and can present as skeletal myopathies, with or without myocardial disease. The underlying mechanisms that prompt disproportionate or even exclusive pathologies in different muscles remain unknown. They nevertheless may involve unique protein isoforms or posttranslational modifications that establish distinct “sarcomeric niches,” which divergently respond to a common insult. The different physiological properties and demands of the muscle types could likewise contribute to the unique sensitivity of each tissue to the mutation.

Our results show that a single MSM *Mhc* allele, resulting in <100% mutant myosin, is sufficient to significantly reduce lifespan. Muscle function is severely compromised as evident from highly reduced jump and flight abilities, even in young 1-day-old flies, which progressively declined. While histological examination of IFMs revealed the presence of fairly well-structured myofibrils in late-stage pupae expressing MSM *Mhc*, they were fewer in number and sarcomeres were smaller than in age-matched transgenic controls. Within hours of adult eclosion, a drastic reduction in apparent myofibril content and order was observed. Thus, in general, myofibrillar degeneration appeared more extreme in young MSM adults compared to pupae. Hallmark ultrastructural filamentous inclusions were also seen within the mutant myofibers, as early as the pupal stage.

Furthermore, we examined the cardiac physiology of heterozygous MSM flies to explore the effects of the mutations on another distinct striated muscle type. This study could not be performed previously using MSM homozygotes, as expression of mutant myosins in the absence of wildtype MHC in all musculature, was lethal. All 3 mutant heterozygotes showed striking cardiac phenotypes. Cardiac myofibrils displayed diffuse striations in confocal micrographs relative to the distinct striations seen in transgenic controls. Moreover, hearts of *Drosophila* expressing E1883K *Mhc* showed extrasarcomeric bodies that were highly immunoreactive with the anti-MHC antibody, suggestive of myosin aggregates in the myocardium (Fig. 5a). Cardiac diameters were markedly smaller with a distinct reduction in fractional shortening and cardiac output. All 3 mutants showed similar severity in the cardiac phenotypes. This was intriguing since, excluding the homozygous E1883K individual previously mentioned, few incidences of cardiac involvement in afflicted humans have been reported. This is not uncommon in orphan diseases, however, as diagnosis requires advanced pathological and genetic testing that is not readily available.

To understand the underlying molecular basis of the muscle phenotypes, we mapped the MSM mutations onto the cryo-EM-derived human cardiac thick filament model (PDB 84L; Dutta *et al.* 2023). The thick filament head arrangement, backbone structure, and protein complement of human cardiac muscle differs from that of *Drosophila* IFMs and heart (Daneshparvar *et al.* 2020; Dutta *et al.* 2023). However, the high degree of conservation of residues between species (Supplementary Fig. 1) in the rod suggests that imperative interactions between myosin and species-specific binding partners in this region are likely evolutionarily preserved. Detailed visualization of the human filament backbone, to appreciate the possible effects of the mutations on the tail, revealed that the introduction of a proline at position 1793 potentially interrupted the normal path of the myosin tails, thus disrupting tail–tail interactions vital to stabilize the filament. This is not surprising as proline residues tend to destabilize and distort α-helical strands owing to their inability to form hydrogen bonds and through steric hindrance (MacArthur and Thornton 1991). Replacing the positively charged Arg1845 with a neutral and bulky Trp at the critical nodes of contact would alter the local surface charge and additionally increase steric hindrance proximally, at tail–tail interfaces, which would be expected to weaken the rigidity of the filament core. The E1883K charge reversal likely disrupts critical points of contact in the highly conserved ACD which would weaken the interactome. Thus, there was no clear unifying mechanism through which these MSM-inducing mutations in the human cardiac thick filament putatively result in disease. The predicted underlying molecular pathology was as varied as the spectrum of clinical presentation. This was unsurprising, as previous studies have shown that mutations in the same myosin residue, while resulting in subtly different molecular phenotypes, could result in divergent diseases (Armel and Leinwand 2010; Liu *et al.* 2024). Interestingly, a recent report of a mouse model for the myosin rod disease Laing distal myopathy showed that substitution of a proline in the tail shifts myosin heads from a super-relaxed state to a disordered relaxed state, which is more capable of ATP hydrolysis and hypercontractility (Buvoli *et al.* 2024). It is feasible that a similar mechanistic shift in myosin head function could account for the partially contracted resting cardiomyocytes, decreased cardiac diameters, and IFM hypercontraction seen in our models of MSM.

We have, therefore, established that even in the heterozygous state, our *Drosophila* MSM models dependably recapitulate disease. Progressive skeletal muscle weakness and myofibrillar architectural alterations, along with characteristic ultrastructural inclusions, were observed. Cardiac structural defects and physiologic dysfunction were also noted in all mutants, with unique underlying disruptions of the thick filament backbone. Thus, despite known differences between *Drosophila* and human MHC and thick filament structures (Daneshparvar *et al.* 2020; Dutta *et al.* 2023), we posit that by destabilizing tail–tail interactions near or within the ACD, the L1793P, R1845W, and E1883K MSM mutations disturb both antiparallel and parallel myosin packing (Dutta *et al*. 2023), and overall thick filament backbone rigidity across species, possibly by analogous or even unique mechanisms. The weakened filament backbone may thus be unable to bear and/or transmit the tension produced by its normally functioning complement of S1 motors or any externally applied forces. Consequently, individual molecules may be released from the filament, or struggle to incorporate, further diminishing backbone and sarcomeric integrity, culminating in myofibrillar and myocyte damage and weakness. Hence, our viable *Drosophila* models, with the potential to express exclusively mutant or 50% mutant protein with retained (but reduced) flight ability, could potentially serve as valuable tools for wide-scale testing of therapeutic approaches to improve muscle performance or ameliorate accumulation of the pathologic aggregates that contribute to the myopathic phenotype.

## Supplementary Material

iyae174_Supplementary_Data

## Data Availability

The authors affirm that all data necessary for verifying the conclusions are present within the article, figures, tables, and Supplementary material. All raw data used for figures can be found in Supplementary Data 1. Table 1 lists all strains examined in this study, which are available upon request. Sequence data are available at GenBank and the accession numbers are listed in Supplementary Fig. 1. Supplementary material available at GENETICS online.
