# Supplementary material for: Dominant myosin storage myopathy mutations disrupt striated muscles in Drosophila and the myosin tail–tail interactome of human cardiac thick filaments
Source: Genetics. 2024 Nov 1;229(1):iyae174. doi: 10.1093/genetics/iyae174 (PMC11708916; doi:10.1093/genetics/iyae174)
Supplement: iyae174_Supplementary_Data [file iyae174_supplementary_data.pdf]

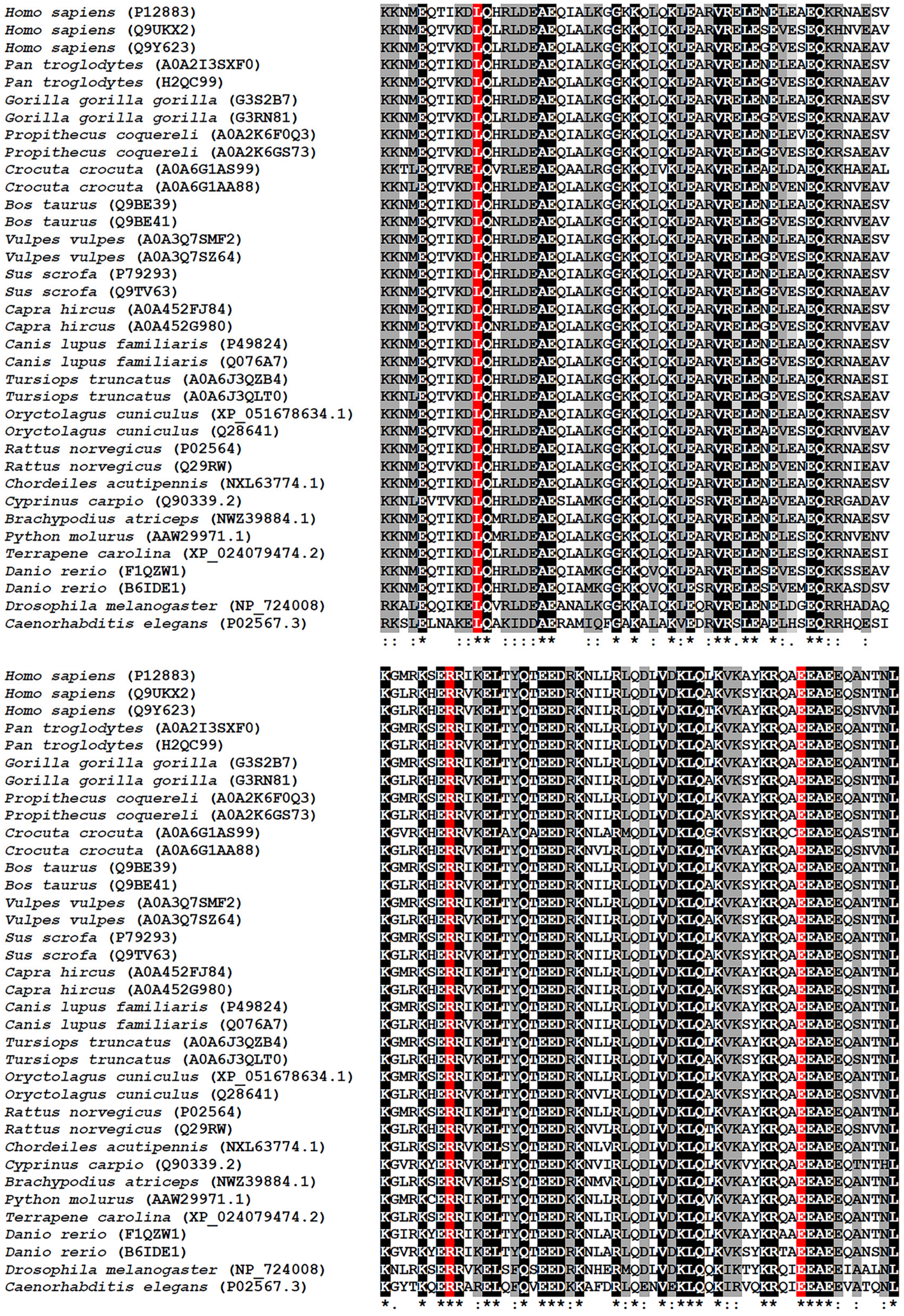
Homo sapiens (P12883) KKNMEQTIKDLQHRLDEAEQIALKGGKKQLQKLEARVRELENELEAEQKRNAESV
Homo sapiens (Q9UKX2) KKNMEQTVKDLQLRLDEAEQLALKGGKKQIQKLEARVRELESEVESEQKHNVEAV
Homo sapiens (Q9Y623) KKNMEQTVKDLQLRLDEAEQLALKGGKKQIQKLEARVRELEGEVESEQKRNAEAV
Pan troglodytes (A0A2I3SXF0) KKNMEQTIKDLQHRLDEAEQIALKGGKKQLQKLEARVRELENELEAEQKRNAESV
Pan troglodytes (H2QC99) KKNMEQTVKDLQLRLDEAEQLALKGGKKQIQKLEARVRELEGEVESEQKRNAEAV
Gorilla gorilla gorilla (G3S2B7) KKNMEQTIKDLQHRLDEAEQIALKGGKKQLQKLEARVRELENELEAEQKRNAESV
Gorilla gorilla gorilla (G3RN81) KKNMEQTVKDLQLRLDEAEQLALKGGKKQIQKLEARVRELEGEVESEQKRNAEAV
Propithecus coquereli (A0A2K6F0Q3) KKNMEQTIKDLQHRLDEAEQIALKGGKKQLQKLEARVRELENELEVEQKRNAESV
Propithecus coquereli (A0A2K6GS73) KKNMEQTVKDLQHRLDEAEQLALKGGKKQIQKLEARVRELEGEVESEQKRSAEAV
Crocuta crocuta (A0A6G1AS99) KKTLEQTVRELQVRLEEAEQAALRGGKKQIVKLEAKVRELEAELDAEQKKHAEAL
Crocuta crocuta (A0A6G1AA88) KKNLEQTVKDLQHRLDEAEQLALKGGKKQIQKLEARVRELENEVENEQKRNVEAV
Bos taurus (Q9BE39) KKNMEQTIKDLQHRLDEAEQIALKGGKKQLQKLEARVRELENELEAEQKRNAESV
Bos taurus (Q9BE41) KKNMEQTVKDLQNRLDEAEQLALKGGKKQIQKLEARVRELEGEVESEQKRNVEAV
Vulpes vulpes (A0A3Q7SMF2) KKNMEQTIKDLQHRLDEAEQIALKGGKKQLQKLEARVRELENELEAEQKRNAESV
Vulpes vulpes (A0A3Q7SZ64) KKNMEQTVKDLQHRLDEAEQLALKGGKKQIQKLEARVRELEGEVESEQKRNAEAV
Sus scrofa (P79293) KKNMEQTIKDLQHRLDEAEQIALKGGKKQLQKLEARVRELENELEAEQKRNAESV
Sus scrofa (Q9TV63) KKNMEQTVKDLQHRLDEAEQLALKGGKKQIQKLEARVRELEGEVESEQKRNAEAV
Capra hircus (A0A452FJ84) KKNMEQTIKDLQHRLDEAEQIALKGGKKQLQKLEARVRELENELEAEQKRNAESV
Capra hircus (A0A452G980) KKNMEQTVKDLQNRLDEAEQLALKGGKKQIQKLEARVRELEGEVESEQKRNVEAV
Canis lupus familiaris (P49824) KKNMEQTIKDLQHRLDEAEQIALKGGKKQLQKLEARVRELENELEAEQKRNAESV
Canis lupus familiaris (Q076A7) KKNMEQTVKDLQHRLDEAEQLALKGGKKQIQKLEARVRELEGEVESEQKRNAEAV
Tursiops truncatus (A0A6J3QZB4) KKNMEQTIKDLQHRLDEAEQIALKGGKKQLQKLEARVRELENELEAEQKRNAESI
Tursiops truncatus (A0A6J3QLT0) KKNLEQTVKDLQHRLDEAEQLALKGGKKQIQKLEARVRELEGEVESEQKRSAEAV
Oryctolagus cuniculus (XP_051678634.1) KKNMEQTIKDLQHRLDEAEQIALKGGKKQLQKLEARVRELENELEAEQKRNAESV
Oryctolagus cuniculus (Q28641) KKNMEQTVKDLQHRLDEAEQLALKGGKKQIQKLEARVRELEAEVESEQKRNVEAV
Rattus norvegicus (P02564) KKNMEQTIKDLQHRLDEAEQIALKGGKKQLQKLEARVRELENELEAEQKRNAESV
Rattus norvegicus (Q29RW) KKNMEQTVKDLQHRLDEAEQLALKGGKKQIQKLEARVRELENEVENEQKRNIEAV
Chordeiles acutipennis (NXL63774.1) KKNMEQTIKDLQLRLDEAEQLALKGGKKQLQKLEARVRELENELEAEQKRNAESV
Cyprinus carpio (Q90339.2) KKNLEVTVKDLQHRLDEAESLAMKGGKKQLQKLESRVRELEAEVEAEQRRGADAV
Brachypodius atriceps (NWZ39884.1) KKNMEQTIKDLQMRLDEAEQLALKGGKKQLQKLEARVRELENELEAEQKRNAESV
Python molurus (AAW29971.1) KKNMEQTIKDLQMRLDEAEQLALKGGKKQLQKLEARVRELENELESEQKRNVENV
Terrapene carolina (XP_024079474.2) KKNMEQTIKDLQLRLDEAEQLALKGGKKQLQKLEARVRELENELESEQKRNAESI
Danio rerio (F1QZW1) KKNMEQTIKDLQHRLDEAEQIAMKGGKKQVQKLEARVRELESEVESEQKKSSEAV
Danio rerio (B6IDE1) KKNMEQTIKDLQHRLDEAEQIAMKGGKKQVQKLESRVRELESEVEMEQRKASDSV
Drosophila melanogaster (NP_724008) RKALEQQIKELQVRLDEAEANALKGGKKAIQKLEQRVRELENELDGEQRRHADAQ
Caenorhabditis elegans (P02567.3) RKSLELNAKELQAKIDDAERAMIQFGAKALAKVEDRVRSLEAELHSEQRRHQESI
:: :* ::** ::::** :: * * : *:* :**.** *:. **:: :
Homo sapiens (P12883) KGMRKSERRIKELTYQTEEDRKNLLRLQDLVDKLQLKVKAYKRQAEEAEEQANTNL
Homo sapiens (Q9UKX2) KGLRKHERRVKELTYQTEEDRKNILRLQDLVDKLQAKVKSYKRQAEEAEEQSNTNL
Homo sapiens (Q9Y623) KGLRKHERRVKELTYQTEEDRKNILRLQDLVDKLQTKVKAYKRQAEEAEEQSNVNL
Pan troglodytes (A0A2I3SXF0) KGMRKSERRIKELTYQTEEDRKNLLRLQDLVDKLQLKVKAYKRQAEEAEEQANTNL
Pan troglodytes (H2QC99) KGLRKHERRVKELTYQTEEDRKNILRLQDLVDKLQAKVKSYKRQAEEAEEQSNTNL
Gorilla gorilla gorilla (G3S2B7) KGMRKSERRIKELTYQTEEDRKNLLRLQDLVDKLQLKVKAYKRQAEEAEEQANTNL
Gorilla gorilla gorilla (G3RN81) KGLRKHERRVKELTYQTEEDRKNILRLQDLVDKLQAKVKSYKRQAEEAEEQSNTNL
Propithecus coquereli (A0A2K6F0Q3) KGMRKSERRIKELTYQTEEDRKNLLRLQDLVDKLQLKVKAYKRQAEEAEEQANTNL
Propithecus coquereli (A0A2K6GS73) KGLRKHERRVKELTYQTEEDRKNILRLQDLVDKLQAKVKSYKRQAEEAEEQSNTNL
Crocuta crocuta (A0A6G1AS99) KGVRKHERRVKELAYQAEEDRKNLARMQDLVDKLQGKVKSYKRQCEEAEEQASTNL
Crocuta crocuta (A0A6G1AA88) KGLRKHERRVKELTYQTEEDRKNVLRLQDLVDKLQTKVKAYKRQAEEAEEQSNVNL
Bos taurus (Q9BE39) KGMRKSERRIKELTYQTEEDRKNLLRLQDLVDKLQLKVKAYKRQAEEAEEQANTNL
Bos taurus (Q9BE41) KGLRKHERRVKELTYQTEEDRKNILRLQDLVDKLQAKVKSYKRQAEEAEEQSNTNL
Vulpes vulpes (A0A3Q7SMF2) KGMRKSERRIKELTYQTEEDRKNLLRLQDLVDKLQLKVKAYKRQAEEAEEQANTNL
Vulpes vulpes (A0A3Q7SZ64) KGLRKHERRVKELTYQTEEDRKNILRLQDLVDKLQAKVKSYKRQAEEAEEQSNTNL
Sus scrofa (P79293) KGMRKSERRIKELTYQTEEDRKNLLRLQDLVDKLQLKVKAYKRQAEEAEEQANTNL
Sus scrofa (Q9TV63) KGLRKHERRVKELTYQTEEDRKNILRLQDLVDKLQAKVKSYKRQAEEAEEQSNTNL
Capra hircus (A0A452FJ84) KGMRKSERRIKELTYQTEEDRKNLLRLQDLVDKLQLKVKAYKRQAEEAEEQANTNL
Capra hircus (A0A452G980) KGLRKHERRVKELTYQTEEDRKNILRLQDLVDKLQAKVKSYKRQAEEAEEQSNTNL
Canis lupus familiaris (P49824) KGMRKSERRIKELTYQTEEDRKNLLRLQDLVDKLQLKVKAYKRQAEEAEEQANTNL
Canis lupus familiaris (Q076A7) KGLRKHERRVKELTYQTEEDRKNILRLQDLVDKLQAKVKSYKRQAEEAEEQSNTNL
Tursiops truncatus (A0A6J3QZB4) KGMRKSERRIKELTYQTEEDRKNLLRLQDLVDKLQLKVKAYKRQAEEAEEQANTNL
Tursiops truncatus (A0A6J3QLT0) KGLRKHERRVKELTYQTEEDRKNILRLQDLVDKLQAKVKSYKRQAEEAEEQSNTNL
Oryctolagus cuniculus (XP_051678634.1) KGMRKSERRIKELTYQTEEDRKNLLRLQDLVDKLQLKVKAYKRQAEEAEEQANTNL
Oryctolagus cuniculus (Q28641) KGLRKHERRVKELTYQTEEDRKNVLRLQDLVDKLQAKVKSYKRQAEEAEEQSNVNL
Rattus norvegicus (P02564) KGMRKSERRIKELTYQTEEDRKNLLRLQDLVDKLQLKVKAYKRQAEEAEEQANTNL
Rattus norvegicus (Q29RW) KGLRKHERRVKELTYQTEEDRKNVLRLQDLVDKLQTKVKAYKRQAEEAEEQSNVNL
Chordeiles acutipennis (NXL63774.1) KGLRKSERRVKELSYQTEEDRKNLVRLQDLVDKLQLKVKAYKRQAEEAEEQANTNL
Cyprinus carpio (Q90339.2) KGVRKYERRVKELTYQTEEDKKNVIRLQDLVDKLQLKVKVYKRQAEEAEEQTNTHL
Brachypodius atriceps (NWZ39884.1) KGLRKSERRVKELSYQTEEDRKNMVRLQDLVDKLQLKVKAYKRQAEEAEEQANTNL
Python molurus (AAW29971.1) KGMRKCERRIKELTYQTEEDKKNLLRLQDLVDKLQVKVKAYKRQAEEAEEQANTNL
Terrapene carolina (XP_024079474.2) KGLRKSERRIKELTYQTEEDRKNLIRLQDLVDKLQLKVKAYKRQAEEAEEQANTNL
Danio rerio (F1QZW1) KGIRKYERRIKELTYQTEEDRKNLARLQDLVDKLQLKVKAYKRAAEEAEEQANTNL
Danio rerio (B6IDE1) KGVRKYERRIKELTYQTEEDRKNLARLQDLVDKLQLKVKSYKRTAEEAEEQANSNL
Drosophila melanogaster (NP_724008) KNLRKSERRVKELSFQSEEDRKNHERMQDLVDKLQQKIKTYKRQIEEAEEIAALNL
Caenorhabditis elegans (P02567.3) KGYTKQERRARELQFQVEEDKKAFDRLQENVEKLQQKIRVQKRQIEEAEEVATQNL
*. * *** :** :* ***:* *:*: *:*** *:: ** ****: : :*

Figure S1: Sequence alignment confirms a high degree of evolutionary conservation of myosin rods. 250 cardiac *MYH7,* skeletal *MYH4*, and *MYH2* sequences were selected from various phyla and aligned using the Clustal Omega (https://www.ebi.ac.uk/jdispatcher/msa/clustalo) multiple sequence alignment program (Sievers *et al.* 2011) to demonstrate the extent of sequence conservation in the myosin distal rod region. Note, for reference, human *MYH7* (P12883) residues 1783 - 1893 are displayed. An (*) indicates positions that have identical residues, a (:) indicates substitution with high structural similarity, and a (.) indicates substitution of low structural similarity. The L1793, R1845 and E1883 residues are shaded red. Accession numbers for each sequence are included.

Sievers F, Wilm A, Dineen DG, Gibson TJ, Karplus K, Li W, Lopez R, McWilliam H, Remmert M, Söding J, Thompson JD, Higgins D. 2011. Fast, scalable generation of high-quality protein multiple sequence alignments using Clustal Omega. Molecular Systems Biology. 7 Article number: 539 doi:10.1038/msb.2011.75.

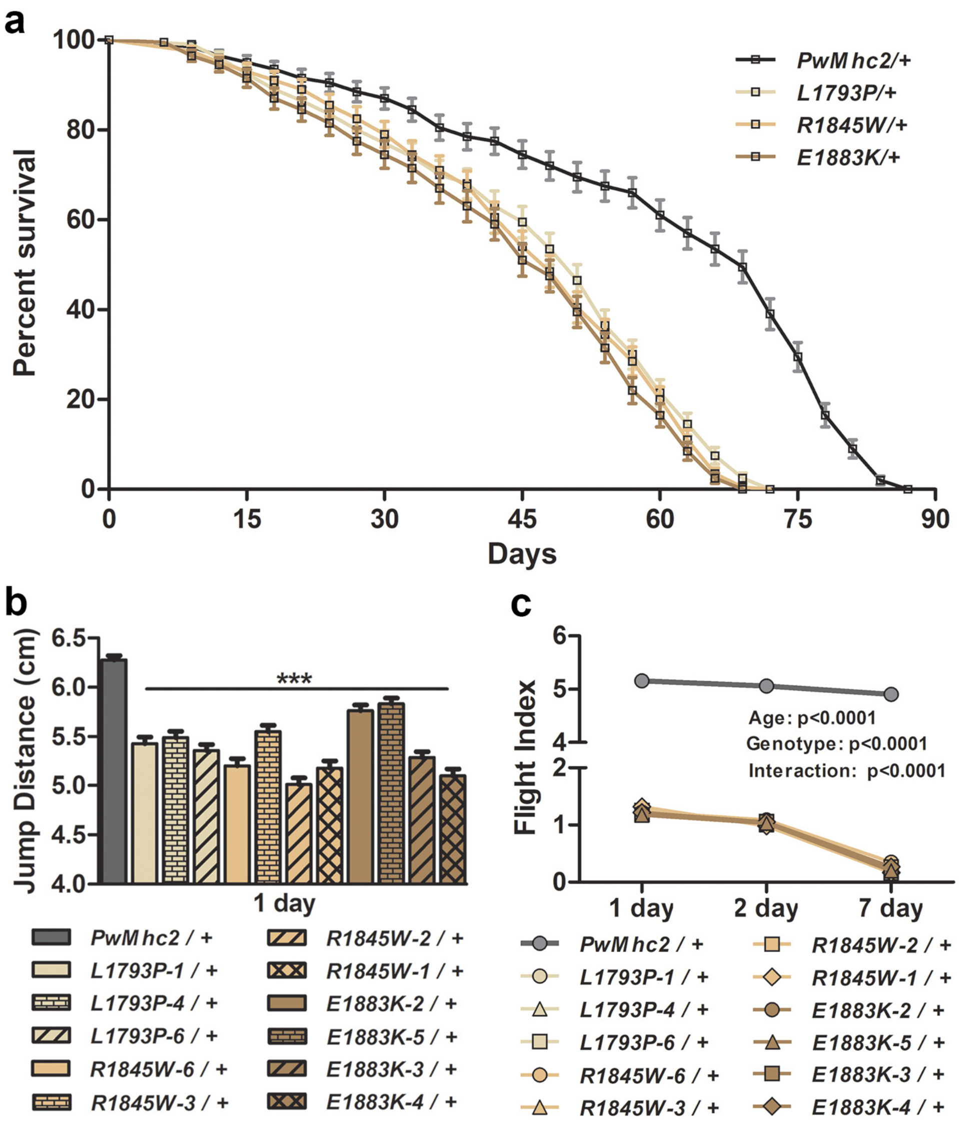

Figure S2: Heterozygous MSM mutations reduce lifespan and skeletal muscle function. (a) Longevity studies of *Mhc⁺/Mhc¹⁰; Mhc*▼ *Drosophila* that possess one mutant and one wildtype *Mhc* copy in the IFMs and jump muscles and one mutant and two endogenous *Mhc* copies in all other muscles. Survival curves revealed a significant reduction in lifespan for all three mutant heterozygotes versus *PwMhc2/+* controls (n = 200 per genotype). Median and maximum lifespans were considerably lower in all mutants (39 - 51 versus 60 days, and 66 - 78 versus 84 days respectively). Two tailed log-rank (Mantel-Cox) tests showed that the decrease in survival was significant between the control and each mutant ($p < 0.0001$). **(b)** Jump distances of heterozygous flies from multiple lines per mutation were comparably reduced relative to the control (***$p < 0.0001$). **(c)** Flight indices of one-, two-, and seven-day-old heterozygotes from the additional lines were reduced relative to age-matched *PwMhc2/+* transgenic controls (genotype $p < 0.0001$; age $p < 0.0001$; interaction $p < 0.0001$).

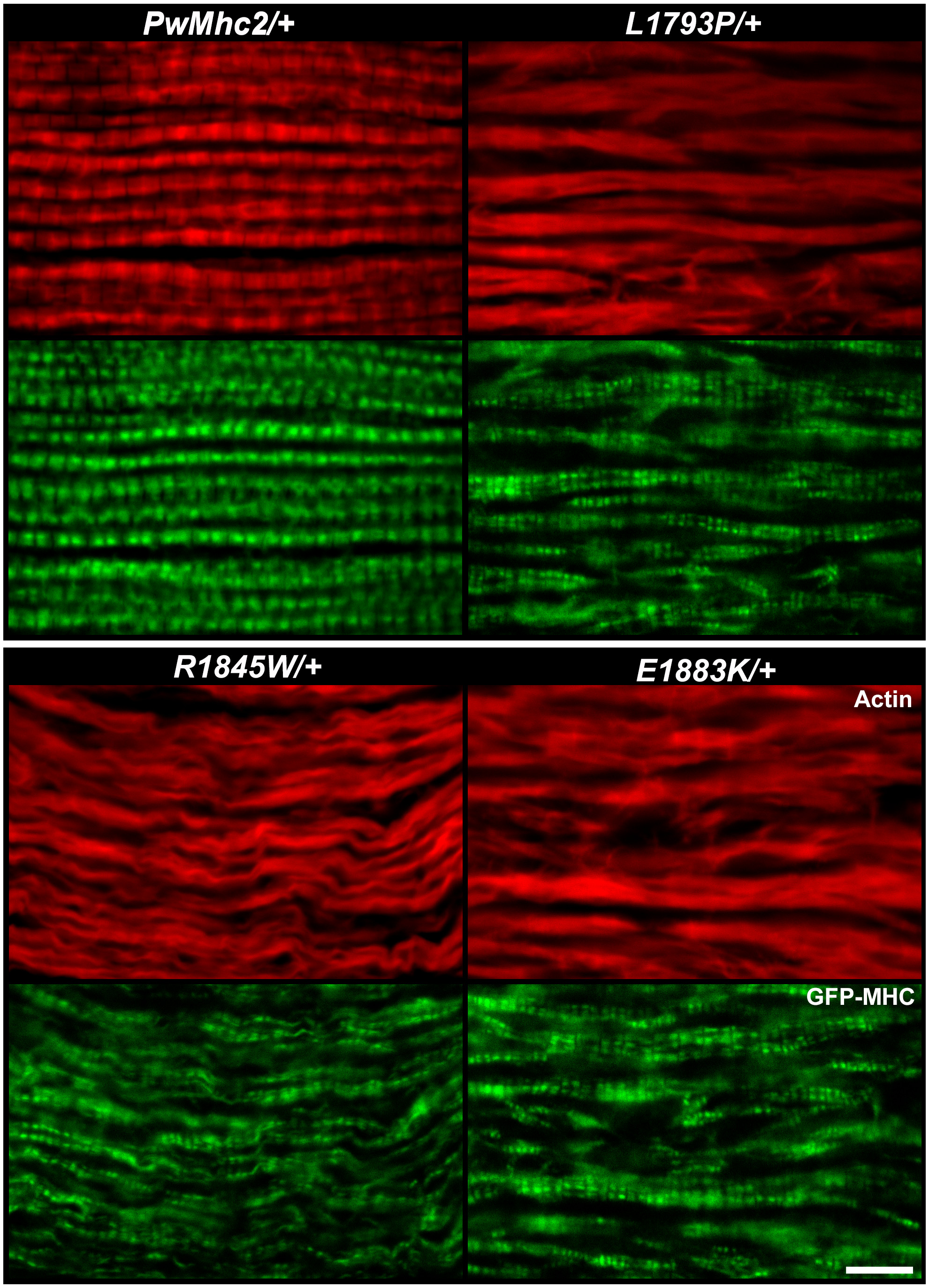

Figure S3: Heterozygous MSM mutations alter myofibrillar and sarcomeric structure. Whole mount IFM confocal images from pupal *PwMhc2* control and mutant heterozygotes. IFMs from fixed and bisected pupae were carefully removed, and myofibrils gently teased apart. Samples were mounted in Vectashield (Vector Laboratories) and imaged on a Leica TCS SPE RGBV confocal microscope at 100X magnification. *PwMhc2/+* pupae showed distinct and uniform IFM myofibrils and ordered sarcomeres with regular I-bands (TRITC-Phalloidin labeled actin) and A-bands (GFP-MHC). All three mutant heterozygotes, however, lacked an ordered arrangement of myofibrils with no distinct thin filaments and few prominent thick filaments. Qualitatively shorter thick filaments were observed. Scale bar = 5 μm.

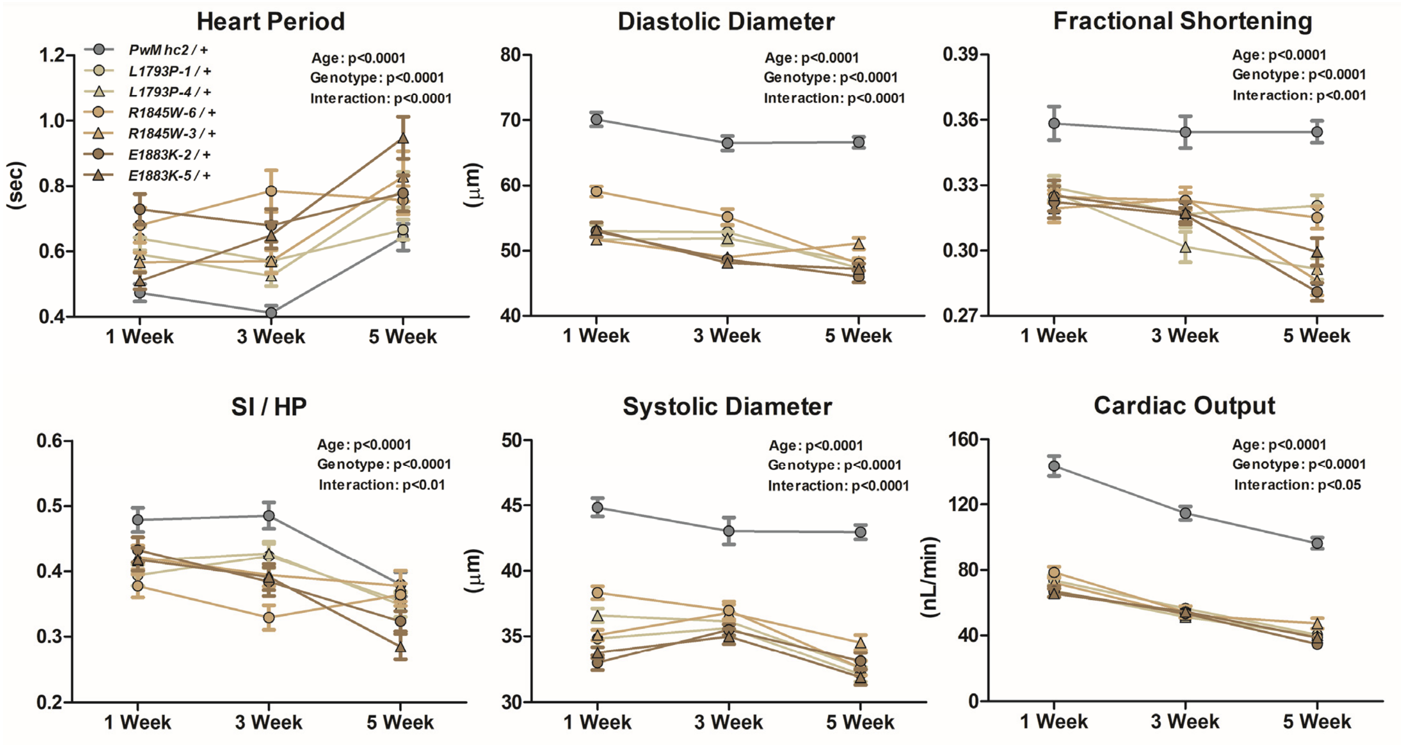

Figure S4: Heterozygous MSM mutations engender restrictive cardiac physiology with reduced cardiac output. Cardiac indices of multiple mutant heterozygous lines indicated severely altered physiology relative to *PwMhc2/+* controls. Longer heart periods with significantly reduced cardiac chamber dimensions (both at systole and diastole) were observed. Reduced fractional shortening and cardiac output were also noted.
